# Size-Dependent Mechanism of Selenium Nanoparticles in Regulating Cadmium Accumulation in the Soil–Rice (*Oryza sativa* L.) System

**DOI:** 10.3390/nano16140897

**Published:** 2026-07-22

**Authors:** Haonan Zhang, Zhangli Lu, Jianhao Tong, Jing Wang, Chendao Ruan, Ziming Xin, Zhenkun Deng, Jiyan Shi

**Affiliations:** 1 Department of Environmental Engineering, College of Environmental and Resource Sciences, Zhejiang University, Hangzhou 310058, China; 2 State Key Laboratory of Soil Pollution Control and Safety, Zhejiang University, Hangzhou 310058, China; 3 State Key Laboratory of Fluid Power and Mechatronic Systems, School of Mechanical Engineering, Zhejiang University, Hangzhou 310058, China; 4 College of Energy Engineering, Zhejiang University, Hangzhou 310027, China

**Keywords:** cadmium, selenium nanoparticles, rice, soil, mechanism, rhizosphere microbial community, size effect

## Abstract

Selenium nanoparticles (Se NPs) have shown potential for regulating cadmium (Cd) accumulation in rice; however, their particle-size-dependent effects in the paddy soil–rice system remain unclear. In this study, a whole-growth-period pot experiment was conducted to investigate the mechanisms of Se NPs with different particle sizes (50, 100, and 200 nm) applied at different rates (20 and 50 mg/kg) in regulating Cd accumulation in rice grown in Cd-contaminated paddy soil. Se NP application significantly altered rhizosphere pH and redox potential, decreased Cd concentrations in soil solution and DTPA-extractable Cd, and increased soil solution Se and KH_2_PO_4_-extractable Se. In the 20 mg/kg treatment, Se NPs promoted the transformation of Cd from exchangeable fractions to Fe–Mn oxide-bound fractions, indicating reduced Cd mobility. Se NPs also enhanced root iron plaque formation and increased the retention of Fe, Cd, and Se on the root surface. XPS analysis showed that 50 and 100 nm Se NPs increased the proportion of Fe(III) in root iron plaque, thereby strengthening Cd adsorption and immobilization at the root–soil interface. In addition, Se NP treatments reshaped rhizosphere microbial communities. The 50 nm treatment showed stronger effects on fungal taxa related to organic matter decomposition and Se activation, whereas the 100 nm treatment more effectively enriched bacterial groups associated with Fe and S cycling, including Thermodesulfobacteriota and Sideroxydans. Among all treatments, 100 nm Se NPs at 20 mg/kg reduced grain Cd from 0.466 to 0.050 mg/kg, representing an 89.27% reduction, while maintaining grain Se at 0.384 mg/kg, which is within the acceptable range for selenium-enriched rice. Overall, 100 nm Se NPs showed the best balance among Cd mitigation, Se biofortification, and ecological safety, suggesting their potential for application in safe rice production in Cd-contaminated paddy soils.

## 1. Introduction

With the rapid development of nanotechnology, the application potential of nanomaterials in agricultural production has received increasing attention. Nanoparticles (NPs) are characterized by a large specific surface area and high surface reactivity, which allow them to play positive roles in improving crop productivity, alleviating environmental pollution, and reducing the risks associated with heavy metals [1,2]. Previous studies have shown that nanomaterials can enhance plant tolerance to cadmium (Cd) stress by regulating gene expression, modifying protein functions, and affecting plant hormones and antioxidant systems [3]. Our previous study also found that pretreatment with 80 mg/L nano-hydroxyapatite effectively regulated rice growth and reduced Cd accumulation in rice seedlings [4]. In addition, owing to their unique surface properties, nanoparticles can act as biostimulants to help plant adapt to heavy metal stress and thereby improve plant stress resistance [5,6].

Selenium (Se) is an essential trace element with important antioxidant, anticancer, and antiviral properties [7]. Exogenous Se can regulate the bioavailability of cadmium (Cd) in the soil–rice system by altering soil physicochemical properties, such as pH, redox potential (Eh), and available phosphorus, thereby inhibiting Cd uptake by rice roots. Root iron plaque is a critical interfacial barrier controlling Cd migration from paddy soil to rice roots. Radial oxygen loss from rice roots can promote the oxidation of Fe^2+^ and the subsequent deposition of Fe/Mn oxides on the root surface. These oxides possess strong adsorption and co-precipitation capacities and can retain metals and metalloids, such as Cd, Pb and As [8]. In addition, exogenous Se may modify soil composition by binding with soil organic matter and mineral phases to form ternary complexes [9], thereby changing the binding modes and adsorption sites of Cd on soil minerals. Se application may also alter the forms and contents of elements such as phosphorus in soil [10], which can further influence Cd speciation and ultimately regulate Cd bioavailability. However, most previous studies have focused on the effects of nanomaterial type and application concentration [11,12], while relatively less attention has been paid to the biological effects induced by differences in nanoparticle size. Under actual flooding–drying conditions, element cycling and energy metabolism in the rice rhizosphere microenvironment are strongly influenced by the rhizosphere conditions of the soil–rice system [13,14]. The exogenous application of Se NPs with different particle sizes may alter the bioavailability of Se and Cd in rhizosphere soil at different growth stages, affect rice biomass accumulation, and further influence microbial community succession and ecological functions in the rhizosphere [15]. Therefore, clarifying the size-dependent behavior of Se NPs throughout the whole growth period of rice is essential for understanding their regulatory effects on Cd accumulation in rice grains.

Previous studies have demonstrated that microorganisms participate in element cycling and energy metabolism in exogenous selenium added Cd-contaminated soils, and that microbial Se release and Cd immobilization contribute to the reduction in Cd accumulation in rice grains [16]. As an important component of soil ecosystems, microorganisms can regulate soil fertility and alleviate heavy metal stress through multiple pathways [17,18]. The application of Se NPs with different particle sizes to Cd-contaminated soils may differentially affect rhizosphere element cycling and microbial community succession during the whole growth period of rice, thereby influencing grain Cd accumulation and rhizosphere antioxidant capacity. In addition, iron plaque on rice roots, which is mainly composed of iron and manganese oxides or hydroxides, acts as an important interface between soil and rice roots [19]. Previous studies have shown that nanoparticles can affect the formation of iron plaque and that this effect may exhibit particle-size dependence [20]. The bioavailability of Se NPs in rice has also been reported to be strongly affected by particle size [21]. Moreover, basal application of Se NPs can activate soil enzymes such as catalase and urease, thereby enhancing rice antioxidant capacity, nitrogen cycling, and material metabolism [17,22]. Exogenous Se application can also recruit beneficial microbial taxa and regulate ecological functions in paddy soils, thus alleviating oxidative stress caused by Cd contamination [23]. Therefore, revealing how Se NPs with different particle sizes regulate the elemental composition of root iron plaque, soil fertility, and rhizosphere microbial community succession in the soil–rice system is critical for elucidating their mechanisms in controlling grain Cd accumulation.

Nanomaterials have emerged as multifunctional platforms in agricultural biotechnology. For example, calcium carbonate nanoparticles can improve plant nutrition and insect pest tolerance, while nanocarriers have been developed for the controlled delivery of nutrients, biopesticides, and other bioactive compounds [24,25]. These studies demonstrate that particle size, surface properties, and release behavior are critical determinants of nanomaterial performance in plant–soil systems. In this study, a pot experiment was conducted to investigate the effects of basal application of Se NPs with different particle sizes and at different concentrations on rice growth and Se and Cd accumulation. The main objectives were to analyze the variation patterns of Se and Cd bioavailability in soil under different treatments, characterize the elemental composition of rice root iron plaque, evaluate soil fertility indicators including nutrient contents and soil enzyme activities, and determine the succession of rhizosphere microbial communities and ecological functions during the whole growth period of rice. These results are expected to clarify the main mechanisms through which the particle-size effect of Se NPs regulates Cd accumulation in the soil–rice system.

## 2. Materials and Methods

### 2.1. Se NP Synthesis and Characterization

Se nanoparticles (Se NPs) were prepared through a chemical reduction method using sodium selenite (Na_2_SeO_3_) and sodium thiosulfate pentahydrate (Na_2_S_2_O_3_·5H_2_O) as precursors, following the protocol reported by Lin and Wang [26] with minor modifications. The morphology and primary particle size of the synthesized Se NPs were examined using scanning electron microscopy (SEM, SU-8010, Hitachi High-Tech Corporation, Tokyo, Japan) and transmission electron microscopy (TEM, H-7650, Hitachi High-Tech Corporation, Tokyo, Japan) [27]. The obtained Se NPs exhibited relatively uniform spherical morphology, with average primary particle sizes of 57.74 ± 26.42, 107.74 ± 32.51, and 207.74 ± 39.11 nm for the nominal 50, 100, and 200 nm Se NP treatments, respectively (Figure S1). The morphology and elemental composition of the Se NPs were characterized by scanning electron microscopy coupled with energy-dispersive X-ray spectroscopy (SEM–EDS) (Figures S10 and S11). Dried Se NP samples were mounted on conductive sample holders for SEM observation, while EDS analysis was used to confirm the elemental composition and spatial distribution of Se. In addition, Raman spectroscopy was employed to further characterize the structural features of the Se NPs based on their characteristic vibrational bands (Figure S12).

Because nanoparticles may undergo aggregation, dispersion, or partial dissolution after being introduced into exposure media, the hydrodynamic diameter and zeta potential were determined to evaluate their effective dispersion status. Dynamic light scattering analysis showed that the dominant intensity-based hydrodynamic diameter peaks of the nominal 50, 100, and 200 nm Se NPs were centered at 77.23, 111.9, and 259.6 nm, respectively. The corresponding zeta potentials were −56.03, −47.34, and −27.99 mV, indicating different colloidal stability among the three particle-size treatments (Figure S2). It should be noted that the primary particle sizes determined by TEM/SEM and the hydrodynamic diameters obtained by DLS represent different physicochemical states of the particles. TEM and SEM were performed on dried samples and mainly reflected the primary particle size, whereas DLS measured the diffusion behavior of particles dispersed in the liquid phase (Table S3). Therefore, the DLS-derived hydrodynamic diameter includes the particle core, hydration layer, electrical double layer, and possible weak aggregation in suspension [28,29]. In addition, the more negative zeta potentials of the 50 and 100 nm Se NPs indicated stronger electrostatic repulsion and better colloidal stability, whereas the lower absolute zeta potential of the 200 nm Se NPs suggested weaker electrostatic stabilization and a greater tendency toward aggregation.

### 2.2. Rice Cultivar and Soil Characteristic

The rice cultivar used in this experiment was Zhongzao 39, and the seeds were purchased from Wuwangnong Seed Industry Co., Ltd. (Hangzhou, China). The tested soil was collected from the surface layer (0–20 cm) of a paddy field at the Yuhang Experimental Base of Zhejiang University Agricultural Experiment Station, located in Jingshan Town, Yuhang District, Hangzhou, Zhejiang Province, China. After collection, the soil samples were placed in turnover boxes and air-dried under ventilated and shaded conditions for approximately 15 days. Stones and plant roots were removed, and the soil was passed through a 2 mm sieve before use. The tested soil was classified as silty loam, and the soil type was paddy soil developed from yellow-red soil. Its basic physicochemical properties are shown in Table 1.

### 2.3. Rice Pot Experiment Design

To provide a clear overview of the experimental workflow, a schematic diagram of the study design, including treatments, sampling stages, and analytical procedures, is presented in Figure S9.

**Rice seedling cultivation.** Rice seeds were rinsed with tap water, surface-sterilized with 30% H_2_O_2_ solution for 30 min, washed three times with deionized water, and then germinated in the dark at 30 °C for 2 days. After germination, the seeds were transferred to hydroponic culture under alternating light and dark conditions: 12 h light (20,000 lx, 30 °C) and 12 h darkness (0 lx, 22 °C), with the relative humidity maintained at 60%. After 7 days of cultivation, the seedlings were transferred to half-strength Kimura nutrient solution (Table S1) and grown for another 7 days, during which the nutrient solution was renewed every 3 days. Fourteen-day-old rice seedlings with uniform growth were then selected and transferred to full-strength Kimura nutrient solution for an additional 7 days. After this cultivation period, seedlings with consistent growth status were selected for the subsequent pot experiment.

**Soil pot cultivation.** The rice pot experiment was conducted in a controlled-climate chamber at the C2-09 Agricultural Experiment Station, Zhejiang University. The growth conditions were set as 16 h light (150 μmol·m^−2^·s^−1^, 28 °C) and 8 h darkness (25 °C), with the relative humidity maintained at 60–70%. A total of 2.5 kg of the pretreated paddy soil described in Section 2.2 was weighed and placed into polyvinyl chloride (PVC) pots with a diameter of 18 cm and a height of 24 cm. In this study, Se NPs were applied to the soil at concentrations of 0, 20, and 50 mg/kg. For each Se NP concentration, three particle sizes, namely 50, 100, and 200 nm, were used, and a Cd-only treatment was set as the control, resulting in seven treatment groups in total. The corresponding amounts of Se NPs were weighed using a stepwise dilution method and thoroughly mixed with the soil to ensure homogeneous distribution in the paddy soil. After mixing, approximately 1.5 L of purified water was added to each PVC pot. The pots were left undisturbed for 2 h to allow the soil to absorb a sufficient amount of water, after which additional purified water was added to maintain a flooding layer of approximately 3 cm. The pots were then continuously flooded for 7 days to stabilize the soil system. During this period, purified water was replenished every 2–3 days to maintain the water layer at approximately 3 cm. After 7 days of soil stabilization, rice seedlings were transplanted into the pots. Meanwhile, rhizosphere soil solution samplers (Rhizon, Wageningen, The Netherlands) and rhizosphere bags were installed in each pot for the subsequent collection of soil solution and rhizosphere soil samples.

### 2.4. Sample Collection and Analytical Methods

**Collection and analysis of rice and rhizosphere soil samples.** During rice cultivation, continuous flooding was maintained before the heading stage for approximately 60 days. From the heading stage to maturation, an alternating flooding–drying water management regime was applied. Rhizosphere soil samples were collected at the initial transplanting stage and throughout the whole growth period. Rice plants were harvested at the maturation stage on day 100 after transplanting. All grains from each plant were collected, and the panicle weight per plant was recorded. The plants were then washed with deionized water, and surface moisture was removed before plant height and whole-plant fresh weight were measured. Subsequently, the roots, stems, leaves, and grains were separated, placed in sealed bags, and stored at −80 °C until further analysis.

At different growth stages, two aliquots of rhizosphere soil solution were collected from each treatment. One aliquot was used for soil solution pH measurement, while the other was stored at 4 °C for subsequent analysis. Soil solution pH was determined using an Orion 5-Star benchtop conductivity multiparameter meter. The soil solution was filtered through a 0.22 μm aqueous membrane filter before analysis. Cadmium concentration in the filtrate was determined by inductively coupled plasma mass spectrometry (ICP-MS, Analytik Jena AG, Jena, Germany), whereas selenium concentration was determined by liquid chromatography–atomic fluorescence spectrometry (LC-AFS 6500, Beijing Haiguang Instrument Co., Ltd., Beijing, China). Soil redox potential (Eh) was measured in situ using the same multiparameter meter. The pH and Eh of rhizosphere soil samples were also determined in situ using a composite electrode (ORP, Model 501, Shanghai INESA Scientific Instrument Co., Ltd., Shanghai, China). Soil available phosphorus (AP), alkali-hydrolyzable nitrogen (AN), and soil organic matter (SOM) were determined using the NaHCO_3_ extraction–molybdenum antimony colorimetric method, alkaline hydrolysis diffusion method, and potassium dichromate volumetric method, respectively. Soil Cd availability was characterized by DTPA-extractable Cd according to GB/T 23739-2009 [30]. The Cd concentration in the filtrate was measured using flame atomic absorption spectrometry (AAS, AAnalyst 900, PerkinElmer, Inc., Waltham, MA, USA). Soil available Se was extracted using the KH_2_PO_4_ extraction method according to NY/T 3420-2019 [31]. Selenium concentration was determined by atomic fluorescence spectrometry. The Tessier sequential extraction method [32] was used to fractionate Cd in rhizosphere soils collected at different rice growth stages into water-soluble/exchangeable Cd (F1), carbonate-bound Cd (F2), Fe–Mn oxide-bound Cd (F3), organic-bound Cd (F4), and residual Cd (F5). The extracts were filtered through 0.22 μm membrane filters, and Cd concentrations were determined using graphite furnace atomic absorption spectrometry (Agilent 240Z AA, Agilent Technologies, Santa Clara, CA, USA). Rice grain samples were digested with a mixed acid solution of HNO_3_ and HClO_4_ at a volume ratio of 4:1, and the concentrations of Se and Cd in the grains were determined using ICP-MS and atomic fluorescence spectrometry, respectively. Soil dehydrogenase activity (DHA), urease (UE) activity, and catalase (CAT) activity were determined using commercial assay kits according to the manufacturer’s instructions, with enzyme activities measured by spectrophotometric methods.

**Determination of elemental composition of root iron plaque.** Rice roots were washed with deionized water to remove residual substances from the root surface, freeze-dried, ground, and stored for further analysis. In this experiment, dithionite–citrate–bicarbonate (DCB) extraction was used to extract Fe, Cd, and Se associated with root iron plaque. A certain amount of freeze-dried rice root sample was placed into a clean conical flask after being rinsed three times with deionized water. Then, 40 mL of freshly prepared DCB extractant, consisting of 0.125 mol/L sodium bicarbonate, 0.03 mol/L sodium citrate, and 0.015 g/mL sodium dithionite, was added. The mixture was shaken at 120 r/min and 25 °C for 1 h to obtain the DCB extract. The DCB extract was filtered through a 0.22 μm aqueous membrane filter to remove impurities. The concentrations of Fe, Se and Cd in the filtrate were measured using flame atomic absorption spectrometry. The elemental contents in root iron plaque were expressed as the concentrations of elements extracted by DCB per kilogram of root dry weight. In addition, selected samples were analyzed by X-ray photoelectron spectroscopy (XPS, Thermo Fisher Scientific Inc., Waltham, MA, USA) for full-spectrum scanning and high-resolution Fe scanning. The chemical forms of Fe were analyzed using Avantage software. The detailed XPS fitting parameters, including peak assignment, redox status, binding energy, FWHM, peak area, relative proportion, and work function, are summarized in Table S2.

### 2.5. Soil Microbial Community Analysis

Based on the results of the pot experiment, rhizosphere soil samples collected at different growth stages from the 20 mg/kg Se NP treatments with particle sizes of 50, 100, and 200 nm, as well as the Cd-only control treatment, were selected for microbial sequencing analysis. The relative abundance and ecological functional characteristics of fungal and bacterial communities under different treatments were analyzed based on the mean values of all samples within each treatment.

Microbial DNA was extracted from rhizosphere soil samples using the E.Z.N.A.^®^ Soil DNA Kit (Omega Bio-tek, Norcross, GA, USA) according to the manufacturer’s instructions. The fungal internal transcribed spacer region (ITS1–1F) was amplified using the primers ITS1F (5′-CTTGGTCATTTAGAGGAAGTAA-3′) and ITS2R (5′-GCTGCGTTCTTCATCGATGC-3′). The V3–V4 region of the bacterial 16S rRNA gene was amplified using the universal primers 341F (5′-barcode-CCTAYGGGRBGCASCAG-3′) and 806R (5′-GGACTACNNGGGTWTCTAAT-3′), where the barcode represents an 8 bp sample-specific sequence. The polymerase chain reaction (PCR) reaction mixture had a total volume of 20 μL, containing 4 μL of 5× FastPfu Buffer, 2 μL of 2.5 mM dNTPs, 0.8 μL of each forward and reverse primer (5 μM), 0.4 μL of FastPfu Polymerase, and approximately 10 ng of template DNA. The PCR amplification program was as follows: initial denaturation at 95 °C for 2 min; 25 cycles of denaturation at 95 °C for 30 s, annealing at 55 °C for 30 s, and extension at 72 °C for 30 s; followed by a final extension at 72 °C for 5 min. The PCR products were checked by 2% agarose gel electrophoresis and purified using the AxyPrep DNA Gel Extraction Kit (Axygen Biosciences, Union City, CA, USA).

The purified PCR products were quantified using a Qubit^®^ 3.0 fluorometer (Invitrogen, Thermo Fisher Scientific Inc., Waltham, MA, USA), and amplicons with different barcodes were pooled in equimolar amounts. After DNA fragmentation using an ultrasonic processor, sequencing libraries were constructed using the NEBNext^®^ Ultra™ DNA Library Prep Kit for Illumina^®^ (New England Biolabs, Inc., Ipswich, MA, USA). Uncyclized molecules were removed to obtain the final libraries. The libraries were submitted to Shanghai Bio-High Technology Co., Ltd. (Shanghai, China), for paired-end sequencing on the Illumina MiSeq high-throughput sequencing platform. Raw FASTQ data were processed using Trimmomatic, and sequences were demultiplexed according to barcode information. PICRUSt2 was used to predict the potential functions of microbial communities. Operational taxonomic units (OTU) data were converted into BIOM format using the make.biom script in Mothur, and taxonomic annotation and metabolic function prediction were performed based on the SILVA database for bacterial 16S rRNA sequences and the UNITE database for fungal ITS sequences.

### 2.6. Statistical Analysis

Each pot treatment included three biological replicates (*n* = 3), which were used for soil physicochemical property, rice growth trait, enzyme activity, Cd/Se fractionation, Cd/Se accumulation, and root iron plaque analyses. Microbial sequencing was performed using three independent rhizosphere soil samples for each selected treatment or growth stage. DLS and zeta potential measurements were performed in triplicate as technical replicates, whereas SEM/EDS, Raman, TEM and XPS analyses were conducted using representative samples.

Data are presented as mean ± standard deviation (SD). Statistical analyses were performed using SPSS 26.0. For soil, rice, enzyme, Cd/Se fractionation, and root iron plaque parameters, statistical comparisons were mainly conducted among the seven treatment groups within the same rice growth stage. One-way analysis of variance (ANOVA) was performed separately for each growth stage, followed by Duncan’s multiple range test when significant treatment effects were detected. Differences were considered statistically significant at *p* < 0.05. No direct statistical comparison across different growth stages was performed for these parameters unless otherwise stated.

Figures were generated using Origin 2022. Rarefaction curves were generated using Mothur v1.21.1, and α-diversity indices, including Chao1, Simpson, and Shannon indices, were calculated to evaluate microbial community richness and diversity. Correlation analyses between microbial communities and environmental factors were performed using the vegan package in R. Growth-stage-related variation in microbial communities was mainly used to describe the temporal succession of rhizosphere microbial communities during rice development.

## 3. Results

### 3.1. Soil Physicochemical Properties and Rice Growth

**Figure 1:**
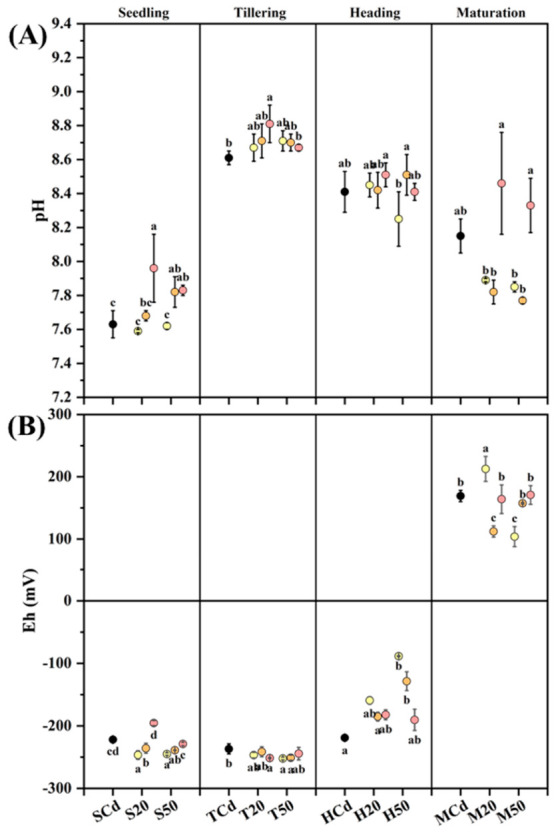
Soil pH (**A**) and Eh (**B**) during different growth stages of rice. “Cd, 20, 50” denotes soil treated with nano-selenium at concentrations of 0, 20, and 50 mg/kg; “S, T, H, M” represent different growth stages; different colors indicate different Se NP particle sizes: yellow, orange, and pink represent 50, 100, and 200 nm Se NPs, respectively. Different letters indicate significant differences between treatments (*p* < 0.05).

The changes in soil solution pH throughout the whole rice growth period are shown in Figure 1A. At the seedling stage, the effect of Se NPs on soil pH began to emerge. The 100 and 200 nm Se NP treatments significantly increased the pH of the soil solution, and the increase became more pronounced with increasing application concentration. With prolonged flooding, the pH values of all treatments increased markedly at the tillering stage, while the effect of Se NP concentration on pH was no longer significant. Among all treatments, the 20 mg/kg 200 nm Se NP treatment showed the most pronounced increase in soil solution pH. After the transition to alternating flooding–drying water management at the heading stage, the pH values of all treatments declined, and the effects of both concentration and particle size became less evident. The 50 mg/kg 50 nm Se NP treatment showed a relatively pronounced decrease in soil solution pH. At the maturation stage, when the soil was completely drained, the effect of application concentration on pH was no longer significant, whereas particle size had a more evident influence on soil pH. Compared with the Cd-only treatment, the 50 and 100 nm Se NP treatments significantly decreased soil pH, while the 200 nm treatment significantly increased soil pH, with a pH value comparable to that observed in the same treatment at the heading stage.

The changes in soil Eh throughout the whole rice growth period are shown in Figure 1B. Soil Eh values were negative during the flooding and alternating flooding–drying stages, including the seedling, tillering, and heading stages, whereas positive Eh values were observed at the maturation stage after complete drainage. At the seedling stage, except for the 20 mg/kg 200 nm Se NP treatment, all Se NP treatments showed lower Eh values than the Cd-only control. A general trend was observed in which smaller Se NPs particle sizes resulted in a more pronounced decrease in soil Eh. With prolonged flooding, the Eh values of all treatments tended to converge at the tillering stage and remained lower than that of the Cd control. At the heading stage, under alternating flooding–drying water management, the high-concentration Se NP treatments (50 mg/kg) showed a trend in which smaller particle sizes led to higher soil Eh values compared with the Cd-only control. Moreover, all Se NP treatments exhibited higher Eh values than the control at this stage. At the maturation stage, the variation in soil Eh was jointly affected by Se NP concentration and particle size. Under the low-concentration treatment (20 mg/kg), the 50 nm Se NP treatment significantly increased soil Eh, whereas the 100 nm Se NP treatment significantly decreased soil Eh, and the 200 nm treatment showed little change. In contrast, under the high-concentration treatment (50 mg/kg), smaller Se NP particle sizes were associated with lower soil Eh values, indicating that the soil redox environment tended to a weakly oxidizing state.

**Figure 2:**
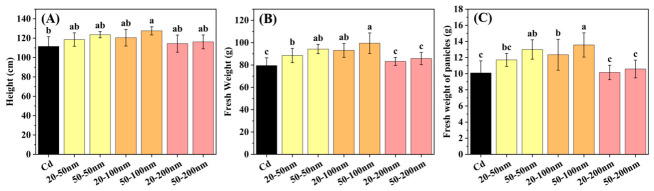
The height (**A**), fresh weight (**B**) and panicle weight per plant (**C**) of rice plants at maturing stage. Letters indicate significant differences between treatments (*p* < 0.05).

Rice plant height, fresh weight, and panicle weight per plant at the maturation stage are shown in Figure 2. At this stage, the 50 and 100 nm Se NP treatments exerted significant positive effects on rice plant height, fresh biomass, and panicle weight. Without considering the potential risk of excessive Se accumulation in the edible parts of rice in this section, the 100 nm Se NP treatment at 50 mg/kg showed the most pronounced yield-promoting effect. Under this treatment, plant height, fresh weight, and panicle weight per plant reached 127.6 cm, 99.5 g, and 13.56 g, respectively, representing increases of 14.44%, 25.16%, and 34.39% compared with the Cd-contaminated control.

### 3.2. Changes in Soil Selenium and Cadmium Availability

**Figure 3:**
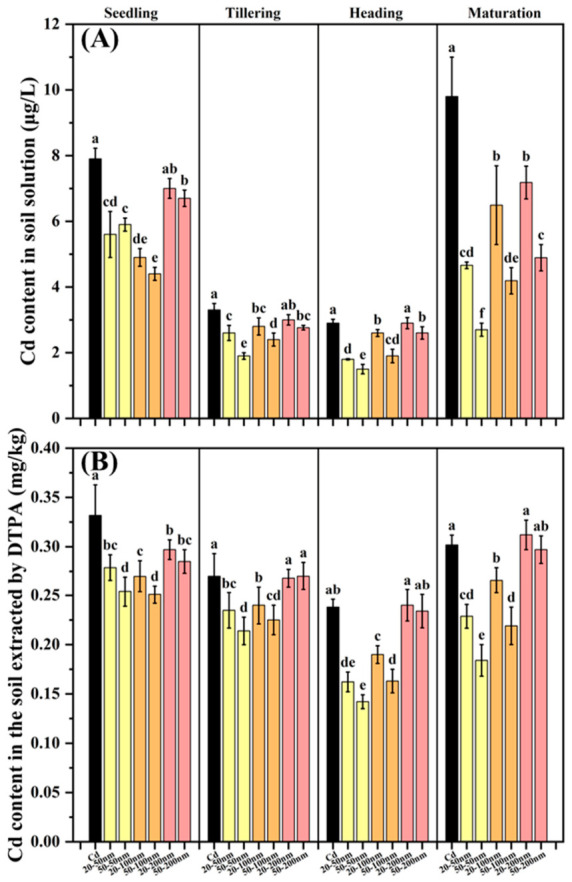
Differences in soil solution Cd content (**A**) and DTPA-extractable Cd content (**B**) at different growth stages of rice. Different letters indicate significant differences among treatments (*p* < 0.05).

The changes in Cd concentration in soil solution are shown in Figure 3A. Soil solution Cd exhibited clear growth-stage-dependent variation. With prolonged flooding, Cd concentrations generally decreased, whereas they increased again at maturation when the soil shifted from flooded to drained conditions [33]. Se NP application significantly reduced soil solution Cd at most growth stages, and this inhibitory effect was particularly evident for the 50 and 100 nm treatments. At the seedling stage, Cd concentration decreased from 7.9 μg/L in the Cd-only control to 5.9 and 4.4 μg/L under the 50 and 100 nm treatments, respectively, while the 200 nm treatment showed a weaker reduction. At the tillering stage, Cd concentrations further decreased in all treatments, but Se NP treatments still maintained lower values than the control. At heading, treatment differences became less pronounced because soil solution Cd was generally low; nevertheless, the 50 and 100 nm treatments still showed significant reductions. At maturation, Se NPs suppressed the drainage-induced increase in soil solution Cd, reducing the value from 9.8 μg/L in the Cd-only treatment to 2.7, 4.19, and 4.89 μg/L under the 50, 100, and 200 nm treatments, respectively. Overall, smaller particle sizes and higher application concentrations tended to produce stronger reductions in soil solution Cd.

The changes in DTPA-extractable Cd were generally consistent with those observed for soil solution Cd (Figure 3B). DTPA-Cd decreased during flooding and increased again at maturation following soil drainage. Se NP treatments significantly reduced DTPA-Cd compared with the Cd-only control, especially under the 50 and 100 nm treatments. At the seedling stage, DTPA-Cd decreased from 0.332 mg/kg in the Cd-only control to 0.254 and 0.251 mg/kg under the 50 and 100 nm treatments, respectively. At tillering, the same decreasing trend was maintained, although the differences among particle sizes were smaller. At heading, DTPA-Cd decreased from 0.238 mg/kg in the Cd-only control to 0.142 and 0.163 mg/kg under the 50 and 100 nm treatments, respectively. At maturation, the Cd-only treatment showed a marked increase in DTPA-Cd, whereas Se NP application inhibited this increase; the 50 and 100 nm treatments remained lower than the control, while the 200 nm treatment showed a weaker effect. These results demonstrate that Se NPs reduced soil Cd availability throughout rice growth, with stronger effects under smaller particle sizes and higher application rates.

**Figure 4:**
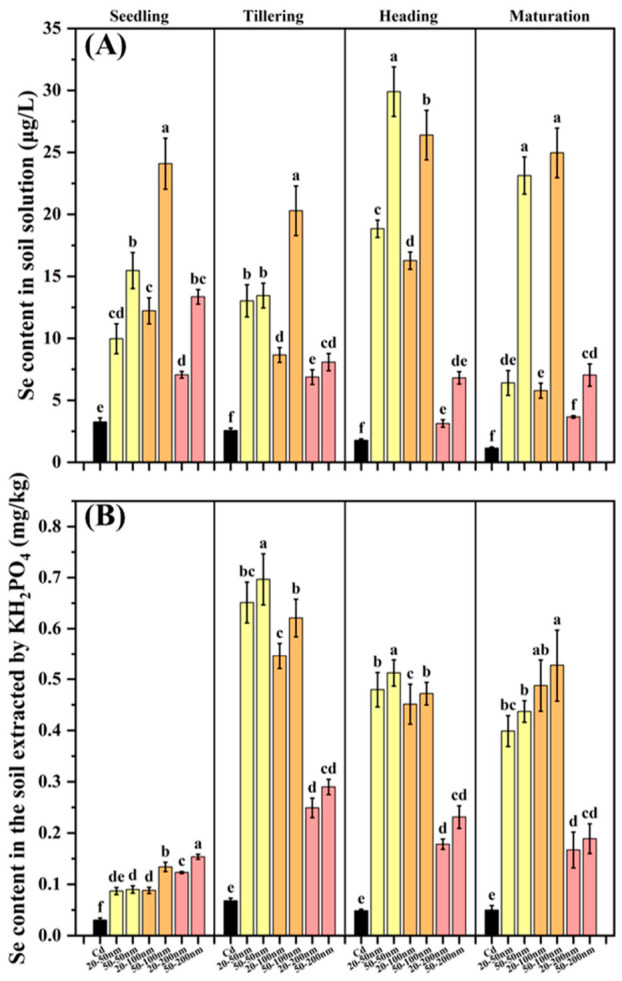
Differences in soil solution Se content (**A**) and KH_2_PO_4_-extractable Se content (**B**) at different growth stages of rice. Different letters indicate significant differences among treatments (*p* < 0.05).

In contrast to Cd, soil solution Se increased significantly after Se NP application and showed distinct stage-dependent variation (Figure 4A). Se concentrations generally increased under flooded conditions and remained higher than those in the Cd-only control throughout the growth period. At the seedling stage, all Se NP treatments increased soil solution Se, with the 50 and 100 nm treatments showing stronger effects than the 200 nm treatment. A similar pattern was maintained at tillering, indicating continuous Se release or activation in the rhizosphere. At heading, soil solution Se reached relatively high levels, with values of 29.905 and 26.401 μg/L under the 50 and 100 nm treatments, respectively, whereas the 200 nm treatment remained much lower. At maturation, soil solution Se decreased slightly in some treatments after drainage but remained significantly higher than that in the Cd-only control. These results indicate that smaller Se NPs were more effective in enhancing Se release or activation in the soil solution.

KH_2_PO_4_-extractable Se showed a pattern similar to that of soil solution Se (Figure 4B). Se NP application significantly increased soil available Se at all growth stages, with stronger responses under high concentration and smaller particle-size treatments. At the seedling stage, the increase in KH_2_PO_4_-extractable Se was already detectable, and the 50–200 nm and 50–100 nm treatments showed relatively high values. At tillering, KH_2_PO_4_-extractable Se increased sharply, reaching 0.697 and 0.621 mg/kg under the 50 and 100 nm treatments at the high application rate, respectively. At heading, available Se decreased slightly but remained much higher than in the Cd-only control. At maturation, the high-concentration 100 nm treatment reached the highest value, while the 50 nm treatment also maintained a high level and the 200 nm treatment remained comparatively low. Together, these results show that Se NPs simultaneously decreased Cd availability and increased Se availability in the rhizosphere, and that both responses were jointly affected by particle size and application concentration.

**Figure 5:**
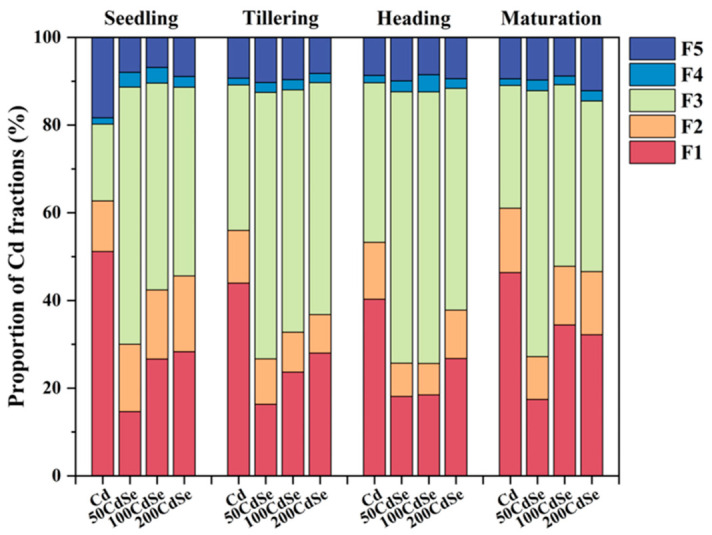
Proportion Cd fractions in rice rhizosphere soil during different growth stages under 20 mg/kg Se NP application. F1: exchangeable fraction; F2: carbonate-binding fraction; F3: Fe-Mn oxide-binding fraction; F4: organic-binding fraction; F5: residual fraction.

To further investigate the mechanism through which Se NPs affected Cd availability in rice rhizosphere soil, the Tessier sequential extraction method was used to analyze Cd fractions in rhizosphere soils treated with different particle sizes of Se NPs at an application rate of 20 mg/kg throughout the whole rice growth period. The results are shown in Figure 5. At the seedling stage, the Cd-only treatment showed the highest proportion of F1 Cd (exchangeable Cd), accounting for 51.17% of the total Cd. In contrast, Se NP treatments significantly reduced the proportion of F1 Cd to 14.66%, 26.64%, and 28.30% in the 50CdSe, 100CdSe, and 200CdSe treatments, respectively. Meanwhile, the proportion of F3 Cd (Fe–Mn oxide-bound Cd) increased markedly from 17.51% in the Cd-only treatment to 58.64% and 47.19% in the 50CdSe and 100CdSe treatments, respectively, indicating that Se NPs promoted the transformation of Cd into Fe–Mn oxide-bound forms. The proportion of F5 Cd (residual Cd) slightly decreased after Se NP application but remained at a certain level. At the tillering stage, the distribution of Cd fractions was generally similar to that observed at the seedling stage. The proportion of F1 Cd in the Cd-only treatment was 43.96%, whereas Se NP treatments significantly decreased this proportion to 16.31% and 23.65% in the 50CdSe and 100CdSe treatments, respectively. In parallel, the proportion of F3 Cd further increased, reaching 60.78% and 55.24% in the 50CdSe and 100CdSe treatments, respectively, which were markedly higher than that in the Cd-only treatment (33.20%). The proportions of F2 Cd (carbonate-bound Cd) and F4 Cd (organic-bound Cd) showed only minor changes among treatments. At the heading stage, the proportion of F1 Cd in the Cd-only treatment was 40.28%, whereas Se NP application significantly reduced it to 18.10% and 18.48% in the 50CdSe and 100CdSe treatments, respectively. F3 Cd remained the dominant fraction, reaching 61.90% and 61.96% in the 50CdSe and 100CdSe treatments, respectively, which were significantly higher than that in the Cd-only treatment (36.39%). Meanwhile, the proportion of F5 Cd slightly increased under Se NP treatments, ranging from approximately 8.52% to 9.93%, suggesting a further transformation of Cd toward more stable fractions. At the maturation stage, the distribution of Cd fractions changed to some extent as the soil gradually shifted from flooded to drained conditions. The proportion of F1 Cd in the Cd-only treatment increased again to 46.34%, while Se NP treatments still significantly reduced this proportion to 17.41%, 34.42%, and 32.19% in the 50CdSe, 100CdSe, and 200CdSe treatments, respectively. The proportion of F3 Cd remained higher in Se NP treatments than in the Cd-only treatment, with values of 27.98%, 60.65%, and 41.39% for the Cd-only, 50CdSe, and 100CdSe treatments, respectively, although it slightly decreased compared with earlier growth stages. In addition, the proportion of F5 Cd increased in some treatments, such as 200CdSe, where it reached 12.17%, indicating an operational redistribution of Cd toward less labile fractions.

Throughout the whole rice growth period, Se NP treatments consistently reduced the proportion of F1 Cd from approximately 40–50% to 14–30% and increased the proportion of F3 Cd from approximately 17–36% to 40–62%. Among the three particle sizes, the 50 nm Se NPs showed the strongest effect. These results indicate that Se NPs reduced Cd bioavailability and mobility mainly by promoting the transformation of Cd from exchangeable forms to Fe–Mn oxide-bound forms in rhizosphere soil.

### 3.3. Characteristics of Selenium and Cadmium Uptake and Accumulation in Rice at the Maturation Stage

**Figure 6:**
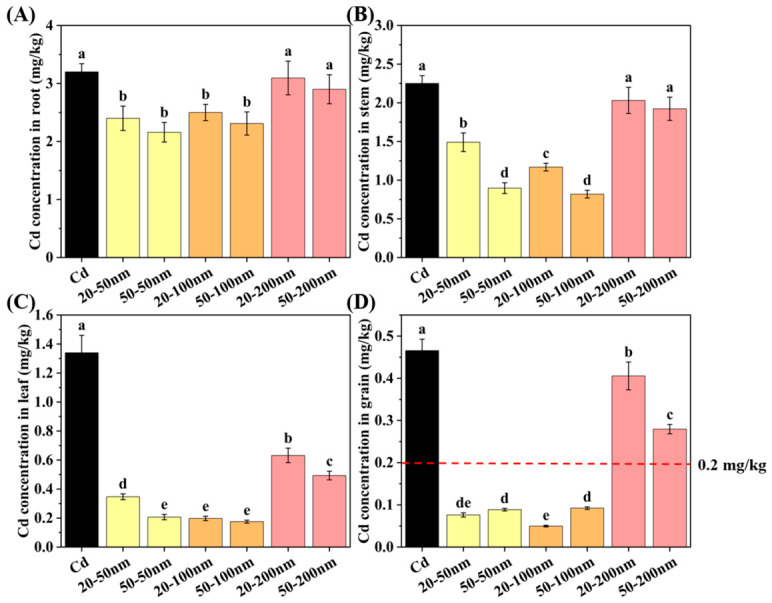
Cd content in different part of rice at the maturation stage. (**A**): root; (**B**): stem; (**C**): leaf; (**D**): grain. Letters indicate significant differences between treatments (*p* < 0.05).

The characteristics of Cd uptake and accumulation in different rice tissues at the maturation stage are shown in Figure 6. The Cd concentrations in roots, stems, leaves, and grains indicated that Se NP application regulated Cd accumulation in rice in a manner jointly affected by application concentration and particle size.

For Cd accumulation in rice roots, the 50 and 100 nm Se NP treatments significantly reduced root Cd concentrations compared with the Cd-only control, and the reduction became more pronounced with increasing application concentration. Among all treatments, the 50 nm Se NP treatment at 50 mg/kg showed a marked decrease in root Cd concentration, reducing it from 3.201 mg/kg in the Cd-only treatment to 2.159 mg/kg, corresponding to a reduction of 32.55%. In contrast, root Cd concentrations in the 200 nm Se NP treatments were not significantly different from those in the Cd-only control, indicating that increasing the application rate of 200 nm Se NPs did not effectively inhibit Cd accumulation in rice roots. For Cd accumulation in rice stems, the effects of Se NP concentration and particle size were generally consistent with those observed in the roots. High-concentration Se NP treatments showed stronger inhibition of Cd translocation to the stems. In particular, the 100 nm Se NP treatment at 50 mg/kg reduced stem Cd concentration from 2.250 mg/kg in the Cd-only treatment to 0.819 mg/kg, corresponding to a reduction of 63.60%. However, the 200 nm Se NP treatments showed no significant difference from the Cd-only control, which was consistent with the hydroponic experiment results and suggested that larger-sized Se NPs were unable to effectively suppress Cd transport through the xylem. For Cd accumulation in rice leaves, all Se NP treatments significantly reduced Cd concentrations compared with the Cd-only control. Compared with the Cd accumulation level of 1.340 mg/kg in the control, the 100 nm Se NP treatment at 50 mg/kg showed the greatest reduction, decreasing leaf Cd concentration to only 0.175 mg/kg, with a reduction of approximately 86.94%. The smaller-sized 50 and 100 nm Se NP treatments still exhibited stronger Cd-inhibiting effects than the 200 nm treatment. For Cd accumulation in rice grains, the 50 and 100 nm Se NP treatments reduced grain Cd concentrations to below the national limit for Cd in cereals and cereal products (<0.2 mg/kg) specified in the National Food Safety Standard for Maximum Levels of Contaminants in Foods (GB 2762-2022) [34]. Notably, the 100 nm Se NP treatment at 20 mg/kg reduced grain Cd concentration from 0.466 mg/kg in the Cd-only treatment to 0.050 mg/kg, corresponding to a reduction of 89.27%.

**Figure 7:**
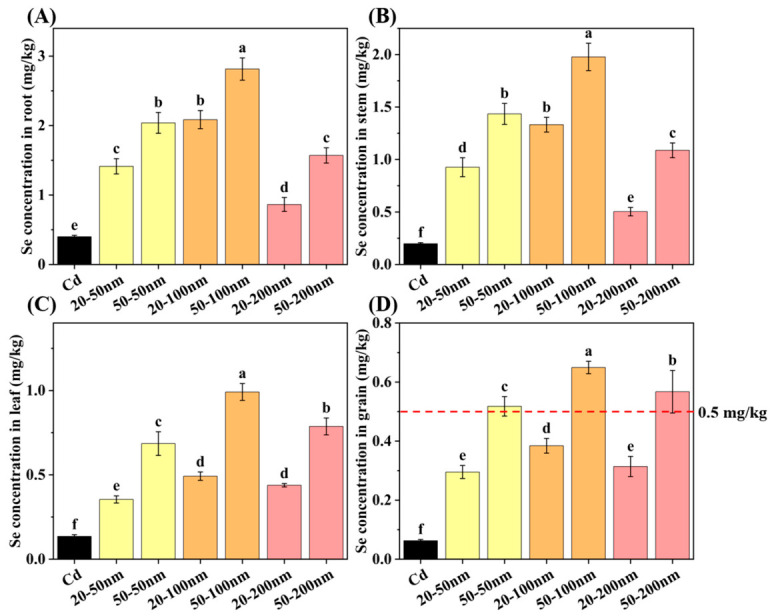
Se content in different part of rice at the maturation stage. (**A**): root; (**B**): stem; (**C**): leaf; (**D**): grain. Letters indicate significant differences between treatments (*p* < 0.05).

The characteristics of Se uptake and accumulation in different rice tissues at the maturation stage are shown in Figure 7. Se accumulation in rice tissues exhibited significant effects from the combination of Se NP application concentration and particle size. For Se accumulation in rice roots, all Se NP treatments significantly increased root Se concentrations compared with the Cd-only control. Among all treatments, the 100 nm Se NP treatment at 50 mg/kg showed the highest root Se concentration, reaching 2.814 mg/kg, which was approximately six times higher than that in the Cd-only treatment (0.400 mg/kg). This result indicates that 100 nm Se NPs had a stronger capacity for rhizosphere adsorption and root uptake under high-concentration application. For Se accumulation in rice stems, the effects of Se NP concentration and particle size were consistent with those observed in the roots. The 100 nm Se NP treatment at 50 mg/kg resulted in a stem Se concentration of 1.977 mg/kg, which was 37.87% and 81.71% higher than those in the 50 nm (1.434 mg/kg) and 200 nm (1.088 mg/kg) treatments at the same application concentration, respectively. For Se accumulation in rice leaves, the effects of Se NP concentration and particle size were also highly consistent with the Se accumulation patterns observed in roots and stems. The 100 nm Se NP treatment at 50 mg/kg showed the highest leaf Se concentration, reaching 0.991 mg/kg, which was 44.67% and 25.92% higher than those in the 50 nm (0.685 mg/kg) and 200 nm (0.787 mg/kg) treatments at the same concentration, respectively. For Se accumulation in rice grains, the grain Se concentrations in all high-concentration Se NP treatments exceeded the threshold specified in the national standard for selenium-enriched paddy rice (GB/T 22499-2025) [35], in which grain total Se content higher than 0.50 mg/kg is considered non-compliant with the selenium-enriched rice standard. For Se accumulation in rice grains, all 50 mg/kg Se NP treatments resulted in grain Se concentrations higher than 0.50 mg/kg. In contrast, grain Se concentrations under all 20 mg/kg Se NP treatments remained below 0.50 mg/kg. Among the 20 mg/kg treatments, the 100 nm Se NP treatment showed the highest grain Se concentration, reaching 0.384 mg/kg. Together with the grain Cd results shown in Figure 6, the 100 nm Se NP treatment at 20 mg/kg reduced grain Cd from 0.466 to 0.050 mg/kg while maintaining acceptable grain Se accumulation.

### 3.4. Changes in Soil Fertility and Elemental Composition of Rice Root Iron Plaque

At the maturation stage, Se NP application significantly affected rhizosphere soil fertility and enzyme activities (Figures S3 and S4). Compared with the Cd-only treatment, Se NPs generally decreased SOM, AN, and UE activity, while increasing AP and CAT activity, with stronger responses observed under smaller particle sizes or higher application concentrations. DHA showed only moderate fluctuations among treatments.

**Figure 8:**
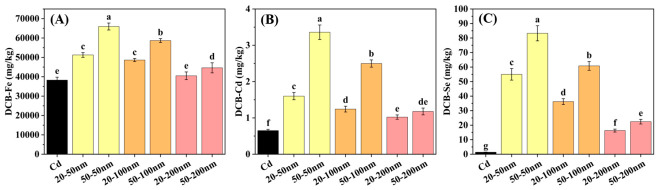
The DCB-Fe (**A**), DCB-Cd (**B**), DCB-Se (**C**) contents in iron plaque at the maturation stage. Letters indicate significant differences between treatments (*p* < 0.05).

Root iron plaque acts as an important barrier for the retention of heavy metals at the root surface. Se NP treatments may affect iron plaque formation by jointly regulating the rhizosphere redox environment, microbial activity, and soil fertility. The contents of Fe, Cd, and Se associated with root iron plaque at the maturation stage are shown in Figure 8. The DCB-extractable Fe (DCB-Fe) results showed that, compared with the Cd-only control, all Se NP treatments except the 20–200 nm treatment significantly increased Fe accumulation in root iron plaque (*p* < 0.05). Among all treatments, the 50–50 nm treatment showed the highest DCB-Fe content, reaching 65,953.82 mg/kg, which was significantly higher than those of the other treatments. This result is consistent with the findings reported by Cheng et al. [21]. Overall, 50 nm Se NPs showed the strongest promoting effect on iron plaque formation. For the same particle size, the high application rate of 50 mg/kg resulted in a greater increase in DCB-Fe than the low application rate of 20 mg/kg. The DCB-extractable Cd (DCB-Cd) results showed that all Se NP treatments significantly enhanced Cd retention in root iron plaque (*p* < 0.05). The highest DCB-Cd content was observed in the 50–50 nm treatment, reaching 3.36 mg/kg. This indicates that Se NPs may strengthen Cd adsorption and immobilization by promoting iron plaque formation, thereby reducing Cd translocation from the root surface into rice tissues. Moreover, smaller particle sizes and higher application concentrations showed stronger effects on Cd retention in root iron plaque. Similarly, the DCB-extractable Se (DCB-Se) results showed that all Se NP treatments significantly increased Se accumulation in root iron plaque (*p* < 0.05). The 50–50 nm treatment also showed the highest DCB-Se content, reaching 83.24 mg/kg.

**Figure 9:**
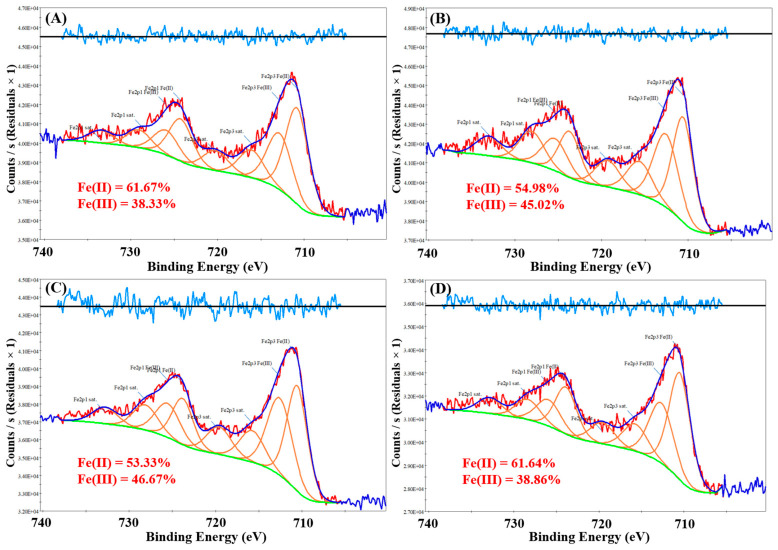
Fe 2p XPS spectrum (8 Scans, 2 m 40.4 s, 400m, CAE 50.0, 0.10 eV) of rice iron plaque in (**A**) Cd treatment, (**B**) 50 nm Se NP treatment, (**C**) 100 nm Se NP treatment, (**D**) 200 nm Se NP treatment at the maturation stage.

To further clarify the effects of Se NPs with different particle sizes on the structure and composition of root iron plaque, XPS analysis was performed on root iron plaque samples collected at the maturation stage from the Cd-only control and the three Se NP treatments at an application rate of 20 mg/kg, with particular focus on the chemical states of Fe (Figure 9). The application rate of 20 mg/kg was selected for this analysis to align with the ecological safety assessment of selenium-enriched paddy rice according to GB/T 22499-2025, while avoiding the potential risk of excessive Se accumulation associated with the 50 mg/kg treatment. High-resolution XPS spectra of Fe 2p showed that Fe in root iron plaque existed mainly as Fe(II) and Fe(III) in all treatments, with Fe(II) being the dominant form. Compared with the Cd-only control, the 50 and 100 nm Se NP treatments significantly decreased the proportion of Fe(II) and correspondingly increased the proportion of Fe(III). In the Cd-only control, Fe(II) and Fe(III) accounted for 61.67% and 38.33%, respectively. In the 50 nm treatment, the proportion of Fe(II) decreased to 54.98%, while that of Fe(III) increased to 45.02%. In the 100 nm treatment, Fe(II) further decreased to 53.33%, and Fe(III) increased to 46.67%. In contrast, the Fe(II)/Fe(III) ratio in the 200 nm treatment was similar to that in the Cd-only control, with Fe(II) and Fe(III) accounting for 61.64% and 38.86%, respectively. These results indicate that smaller Se NPs, particularly the 50 and 100 nm particles, significantly regulated the Fe valence-state composition of root iron plaque by promoting the transformation of Fe(II) to Fe(III), thereby increasing the relative proportion of Fe(III). Fe(III) oxides are key active components of root iron plaque and generally possess stronger adsorption and complexation capacities for heavy metals. Therefore, the increased proportion of Fe(III) could enhance the Cd adsorption and immobilization capacity of root iron plaque, reducing Cd translocation into rice tissues. These findings further confirm that Se NPs reduce Cd accumulation in rice partly by strengthening the root iron plaque barrier, with smaller particle sizes showing more pronounced regulatory effects.

### 3.5. Changes in Rhizosphere Microbial Community Structure and Ecological Functions

To further evaluate the effects of Se NPs on rhizosphere microbial communities, mature rhizosphere soil samples from the low-concentration Se NP treatments suitable for safe rice production were selected for amplicon sequencing. Four treatments, namely MCd, M50, M100, and M200, were analyzed under the 20 mg/kg application rate. A total of 653,148 fungal and 576,480 bacterial valid sequences were obtained from 12 soil samples. NMDS analysis showed that Se NP application altered both fungal and bacterial community structures compared with the Cd-only treatment (Figure S5). The 50 and 100 nm treatments were more clearly separated from the Cd-only treatment, whereas the bacterial community under the 200 nm treatment was relatively close to that of the Cd-only control, suggesting a weaker effect of larger-sized Se NPs on mature rhizosphere bacterial communities.

**Figure 10:**
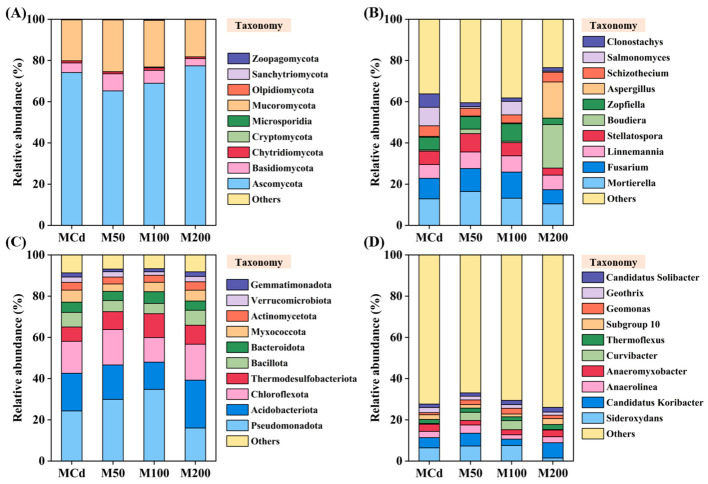
Relative abundance of soil microorganisms in treatment groups with different particle sizes of Se NPs at the maturation stage. Relative abundance of fungal community at (**A**) phylum level (top 10) and (**B**) genus level (top 10); Relative abundance of bacterial community at (**C**) phylum level (top 10) and (**D**) genus level (top 10).

The relative abundances of fungal and bacterial communities under different Se NP particle-size treatments are shown in Figure 10. At the fungal phylum level, Ascomycota, Mucoromycota, and Basidiomycota were the dominant groups. Compared with the MCd treatment, the 50 and 100 nm Se NP treatments decreased the relative abundance of Ascomycota from 74.15% to 65.26% and 68.96%, respectively, while increasing Basidiomycota from 4.65% to 8.28% and 6.25%, respectively. At the genus level, *Mortierella* increased from 12.86% in MCd to 16.47% and 13.18% in M50 and M100, respectively, whereas it decreased to 10.43% in M200. In addition, *Zopfiella* increased from 6.16% to 8.46% in M100, and *Stellatospora* showed an increasing trend under the smaller-sized Se NP treatment but decreased under the 200 nm treatment. At the bacterial phylum level, Pseudomonadota, Acidobacteriota, Chloroflexota, and Thermodesulfobacteriota were the dominant groups. Compared with MCd, the relative abundance of Pseudomonadota increased from 24.32% to 29.92% and 34.78% in the M50 and M100 treatments, respectively, but decreased to 16.02% in M200. In contrast, Acidobacteriota decreased under the 50 and 100 nm treatments but increased under the 200 nm treatment. Notably, Thermodesulfobacteriota increased from 6.95% in MCd to 11.57% in M100, representing the most pronounced increase among the dominant bacterial phyla. At the genus level, *Sideroxydans* increased from 6.39% in MCd to 7.30% and 7.55% in M50 and M100, respectively, but decreased to 1.47% in M200. Similarly, *Curvibacter* increased from 0.32% in MCd to 3.97% and 4.40% in M50 and M100, respectively, but decreased to 0.21% in M200. These results indicate that 50 and 100 nm Se NPs more strongly regulated microbial taxa associated with Fe cycling and C/N cycling than 200 nm Se NPs.

**Figure 11:**
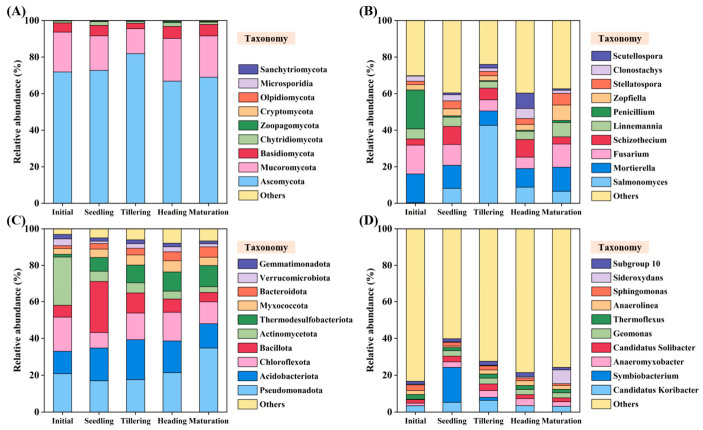
Relative abundance of soil microorganisms in 100 nm Se NP treatment. Relative abundance of fungal community at (**A**) phylum level (top 10) and (**B**) genus level (top 10); Relative abundance of bacterial community at (**C**) phylum level (top 10) and (**D**) genus level (top 10).

Because the 100 nm Se NP treatment at 20 mg/kg showed the best balance between grain Cd reduction and Se biofortification, microbial community succession under this treatment was further analyzed across the rice growth period. A total of 816,435 fungal and 720,600 bacterial valid sequences were obtained from 15 soil samples collected at five growth stages. Rarefaction curves and species accumulation curves indicated sufficient sequencing depth and representative sampling (Figure S6). The α-diversity indices showed stage-dependent changes in fungal and bacterial richness and diversity under the 100 nm Se NP treatment (Figure S7), suggesting that Se NP application exerted a growth-stage-dependent influence on rhizosphere microbial communities. The temporal changes in microbial community composition under the 100 nm Se NP treatment are shown in Figure 11. For fungi, Ascomycota, Mucoromycota, and Basidiomycota were the dominant phyla throughout the growth period. At the genus level, *Salmonomyces*, *Fusarium*, *Schizothecium*, *Zopfiella*, and *Mortierella* were the dominant taxa. Ascomycota peaked at the tillering stage, while *Mucoromycota* reached its highest abundance at the heading stage. *Salmonomyces* increased sharply at the tillering stage and then declined, whereas *Fusarium* and *Schizothecium* remained relatively stable across the growth period. *Zopfiella* increased markedly at the maturation stage, indicating that the fungal community underwent the strongest shift at the tillering stage and gradually stabilized after heading. For bacteria, Pseudomonadota, Acidobacteriota, Chloroflexota, and Thermodesulfobacteriota were the dominant phyla under the 100 nm Se NP treatment. These bacterial groups are closely associated with rhizosphere C, N, Fe, and S cycling. At the genus level, *Symbiobacterium*, *Anaeromyxobacter*, *Geomonas*, *Candidatus Koribacter*, and *Candidatus Solibacter* were the dominant taxa. Notably, the relative abundance of *Sideroxydans* increased from 0.37% at the initial stage to 7.53% at the maturation stage, indicating a strong microbial response related to Fe cycling after Se NP application. Overall, bacterial community composition changed most strongly at the seedling stage and gradually stabilized after tillering. Functional prediction further suggested that fungal and bacterial functional groups related to organic matter decomposition, Fe respiration, nitrate respiration, and nitrate reduction varied across the growth period (Figure S8), supporting the role of Se NPs in regulating rhizosphere element cycling and microbial ecological functions.

## 4. Discussion

### 4.1. Effects of Nano-Selenium on Selenium and Cadmium Availability and Uptake Accumulation in the Soil–Rice System

The results indicate that Se NPs regulated Cd accumulation in the paddy soil–rice system through multiple coupled processes rather than a single pathway. Instead of acting only as a direct Cd-immobilizing amendment, Se NPs appeared to function as a rhizosphere regulator by simultaneously modifying Cd/Se availability, redox-related Cd fraction redistribution, root-surface barrier formation, and plant Cd translocation. These results suggest that Se NPs exerted continuous inhibitory effects on both soil Cd bioavailability and plant Cd uptake. Compared with the 200 nm treatment, the 50 and 100 nm Se NP treatments showed stronger Cd-reducing effects, with the 100 nm treatment achieving a better balance between grain Cd reduction and Se ecological safety. The 50 mg/kg Se NP treatments were not considered preferable primarily because they caused excessive Se accumulation in rice grains. Although this application rate promoted Se uptake, grain Se concentrations exceeded the acceptable range for selenium-enriched rice, which limits its practical suitability for safe rice production. Recent studies have shown that the effects of Se NPs are concentration dependent, with appropriate doses promoting barley seedling growth, whereas higher exposure levels may reduce beneficial effects and increase potential phytotoxicity risks [36].

The regulation of rhizosphere pH and Eh provided an important physicochemical basis for Cd immobilization [37,38,39]. The contrasting effects of Se NP particle size on rhizosphere pH and Eh may be associated with size-dependent differences in Se transformation, colloidal behavior, and interactions with soil components. Previous studies showed that 200 nm Se NPs exhibited a higher transformation rate and generated more available Se than smaller particles in a soil–wheat system, whereas smaller Se NPs were more strongly associated with soil organic matter [40]. Particle size can also affect Se NP stability, mobility, and bioavailability in soil–plant systems [41]. However, our paddy soil–rice system showed higher soil solution and KH_2_PO_4_-extractable Se concentrations under the 50 and 100 nm treatments than under the 200 nm treatment, which may be related to the flooded–drained conditions of paddy soils, where water management strongly regulates pH, Eh, Fe(II), dissolved organic carbon, microbial processes, and Se mobility [42]. Therefore, the divergent pH and Eh responses among particle sizes likely reflect the combined effects of size-dependent Se transformation and redistribution under dynamic paddy-soil conditions. Changes in soil Cd and Se availability further confirmed the particle-size-dependent effects of Se NPs [43]. Soil solution Cd and DTPA-Cd generally decreased under flooding and increased again after drainage, whereas Se NP treatments significantly suppressed this increase, especially at the heading and maturation stages. Tessier sequential extraction showed that Se NPs reduced the proportion of exchangeable Cd and increased Fe–Mn oxide-bound Cd, indicating an operational redistribution of Cd from labile fractions toward less mobile Fe–Mn oxide-associated fractions. Meanwhile, soil solution Se and KH_2_PO_4_-extractable Se increased after Se NP application, suggesting enhanced Se activation and supply. Therefore, Se NPs simultaneously decreased Cd availability and increased Se availability in the rhizosphere, providing a dual mechanism for Cd mitigation and Se biofortification.

Se NPs also altered rhizosphere nutrient cycling and biochemical activity. At the maturation stage, Se NP treatments increased available phosphorus and catalase activity, while decreasing alkali-hydrolyzable nitrogen and urease activity. These changes suggest that Se NPs may promote phosphorus activation, moderate nitrogen turnover, and enhance antioxidant-related soil responses, thereby helping to alleviate Cd-induced stress in the soil–rice system [44]. Root iron plaque was a key interface linking rhizosphere regulation with reduced Cd uptake. Se NP treatments generally increased DCB-extractable Fe, Cd, and Se in root iron plaque, indicating enhanced plaque formation and stronger retention of Cd and Se at the root surface [45]. The Fe plaque barrier likely served as an important intermediate mechanism connecting Se NP-induced rhizosphere redox regulation with reduced Cd translocation in rice. XPS analysis further showed that 50 and 100 nm Se NPs increased the proportion of Fe(III) in root iron plaque, whereas the 200 nm treatment showed little difference from the Cd control. During rice growth, Se NPs altered soil solution pH and Eh in a particle-size and concentration-dependent manner. Soil Eh remained negative under flooded conditions and became positive after drainage, reflecting the typical redox transition of paddy soils. Such redox heterogeneity may promote localized oxidized zones around rice roots, facilitating Fe(II) oxidation to Fe(III) and subsequent iron plaque formation, thereby creating favorable conditions for Cd immobilization and root-surface interception [46]. Since Fe(III) oxides have strong adsorption and complexation capacities for heavy metals, a higher Fe(III) proportion may increase the number of reactive surface sites for Cd retention and reduce the direct entry of Cd into root tissues. These results suggest that smaller Se NPs mitigate Cd accumulation partly by strengthening the Fe plaque barrier.

Overall, 50 nm Se NPs showed stronger effects on Cd immobilization and iron plaque formation, but they also promoted Se activation more strongly, which may increase the risk of excessive grain Se under high application rates. In contrast, 100 nm Se NPs effectively reduced Cd availability, inhibited Cd translocation, and maintained grain Se within the selenium-enriched rice threshold at 20 mg/kg. Meanwhile, the 200 nm particles may have lower effective reactivity and weaker rhizosphere regulation. The 100 nm particles may therefore represent an intermediate size with sufficient activity for Cd mitigation but lower risk of excessive Se accumulation. Therefore, under the present experimental conditions, 100 nm Se NPs represented the most suitable particle size for balancing Cd reduction, Se biofortification, and ecological safety.

### 4.2. Effects of Nano-Selenium on Rhizosphere Microbial Community

Rhizosphere microorganisms are an important biological interface linking exogenous nanomaterials, soil element cycling, and plant stress responses [47,48]. The results showed that Se NP application altered both fungal and bacterial community structures in the rice rhizosphere, and this effect was clearly particle-size dependent. This suggests that Se NPs may act as particle-size-dependent ecological filters in the rhizosphere, selectively favoring microbial groups adapted to altered Se availability, Cd stress, redox conditions, and nutrient status. Compared with the Cd-only control, the 50 and 100 nm treatments caused stronger shifts in microbial community composition, whereas the 200 nm treatment was closer to the Cd control, especially for bacterial communities. This indicates that smaller and medium-sized Se NPs had stronger regulatory effects on rhizosphere microbial structure.

The temporal changes under the 100 nm treatment further suggested that microbial responses to Se NPs were growth-stage dependent. Fungal richness was higher at the seedling and heading stages, whereas bacterial richness and diversity were more pronounced at the tillering stage. Such stage-dependent succession may be driven by dynamic changes in root oxygen release, root exudate composition, flooding–drying conditions, and element cycling during rice growth [49]. Therefore, the tillering and heading stages may represent sensitive periods for microbial responses to Se NP-mediated rhizosphere regulation. Fungal community changes were mainly associated with organic matter decomposition and Se activation. Ascomycota, Mucoromycota, and Basidiomycota were the dominant fungal phyla. The 50 and 100 nm treatments increased the relative abundance of *Mortierella*, *Zopfiella*, and *Stellatospora* compared with the Cd control, while the 200 nm treatment showed weaker or opposite responses. These taxa are commonly involved in organic matter decomposition, organic acid secretion, and nutrient transformation [50,51]. Their enrichment may promote the decomposition of organic residues and the release or reactivation of Se in soil, thereby contributing to the increase in soil solution Se and KH_2_PO_4_-extractable Se. Under the 100 nm treatment, the peak of Ascomycota at the tillering stage was consistent with the marked increase in soil available Se, suggesting that fungal succession may be associated with Se activation and Se supply. In contrast, bacterial community changes may alter the potential functional profiles associated with Fe, S, and N cycling in the rhizosphere. At the maturation stage, the 50 and 100 nm treatments increased the relative abundance of Pseudomonadota, Thermodesulfobacteriota, and the Fe-oxidizing genus *Sideroxydans*, whereas the 200 nm treatment did not show similar enrichment. The increase in *Sideroxydans* was consistent with the enhanced DCB-Fe and DCB-Cd contents and the higher Fe(III) proportion in root iron plaque, indicating that Fe-related bacteria may have participated in Fe^2+^ oxidation, iron plaque formation, and Cd immobilization at the root surface [52]. Meanwhile, the enrichment of Thermodesulfobacteriota and other anaerobic functional groups suggests that Se NPs may also strengthen sulfur cycling, which could potentially reduce Cd bioavailability through sulfide formation and precipitation [53].

Overall, Se NPs regulated rhizosphere microbial communities through two linked ecological processes: fungal-mediated Se activation and bacterial-mediated element cycling and stress regulation. The 50 nm treatment appeared to be more effective in promoting fungal-related responses, while the 100 nm treatment showed more balanced regulation of bacterial groups associated with Fe and S cycling. This microbial response was consistent with enhanced catalase activity, reduced urease activity, lower grain Cd, and safe grain Se accumulation. Therefore, 100 nm Se NPs may provide a more coordinated microbial mechanism for balancing Cd mitigation, Se biofortification, and rhizosphere ecological stability. Future metagenomic, metatranscriptomic, enzyme-level, and isotope-tracing analyses are needed to verify the specific microbial pathways involved in Se transformation, Fe/S cycling, and Cd immobilization.

### 4.3. Limitations and Future Perspectives

Although this study provides evidence for the particle-size-dependent regulation of Cd accumulation by Se NPs in the soil–rice system, several limitations should be acknowledged. First, the experiment was conducted under controlled pot conditions using one paddy soil type and one rice cultivar. Therefore, the observed effects may not fully represent the complexity of field environments, where soil heterogeneity, seasonal variation, irrigation regimes, and cultivar differences may influence the behavior and effectiveness of Se NPs. Future multi-site and multi-season field trials are needed to verify the agronomic feasibility and stability of the 100 nm Se NP treatment.

Second, Cd fractionation was evaluated using the Tessier sequential extraction method. Because this method provides operationally defined fractions, the observed decrease in exchangeable Cd and increase in Fe–Mn oxide-bound Cd should be interpreted as an operational redistribution of Cd rather than direct evidence of mineral-phase transformation. Future studies using X-ray absorption spectroscopy, micro-XRF, or other in situ spectroscopic techniques would help clarify the actual Cd binding environments in the rhizosphere and root iron plaque.

Third, microbial functions were mainly inferred from amplicon sequencing and PICRUSt2-based functional prediction. Although PICRUSt2 provides useful information on the potential functional profiles of microbial communities, it predicts functional potentials based on taxonomic composition rather than directly measuring gene abundance, gene expression, or microbial metabolic activity. Therefore, the predicted enrichment of functions related to Se transformation, Fe/S cycling, nitrogen metabolism, and stress responses should be interpreted as potential ecological roles rather than direct functional evidence. Future studies should combine metagenomics, metatranscriptomics, metabolomics, enzyme assays, and isotope-tracing approaches to directly verify the microbial pathways involved in Se activation, Fe/S cycling, and Cd immobilization in the rhizosphere.

Finally, the long-term fate and environmental safety of Se NPs in paddy soils remain to be further evaluated. Nanoparticle aging, transformation, Se speciation, leaching risk, and potential effects on non-target soil organisms should be systematically assessed before field-scale application. In addition, the optimal application rate, timing, and method of Se NPs should be further optimized to achieve stable Cd mitigation, safe Se biofortification, and minimal ecological risk.

## 5. Conclusions

This study demonstrated that Se NPs regulated Cd accumulation in the Cd-contaminated paddy soil–rice system in a clear particle-size-dependent manner. Among the tested treatments, 100 nm Se NPs at 20 mg/kg showed the best overall performance, reducing grain Cd from 0.466 to 0.050 mg/kg, corresponding to an 89.27% reduction, while maintaining grain Se at 0.384 mg/kg, which is within the appropriate range for selenium-enriched rice. The results indicate that, under the present experimental conditions, the 50 and 100 nm Se NP treatments effectively reduced Cd accumulation in rice grains and therefore showed potential application value. In contrast, even increasing the application rate of 200 nm Se NPs failed to reduce grain Cd concentrations to the food safety standard.

The proposed mechanism involves the coordinated regulation of rhizosphere Cd/Se availability, Cd fraction distribution, root iron plaque formation, and microbial community structure. Se NPs decreased soil solution Cd, DTPA-extractable Cd, and exchangeable Cd while increasing soil available Se. The 50 mg/kg Se NP treatment caused grain Se concentrations to exceed the acceptable range for selenium-enriched rice, indicating a potential food safety risk. Under the 20 mg/kg treatment, Se NPs promoted an operational redistribution of Cd from exchangeable fractions toward Fe–Mn oxide-bound fractions, indicating reduced Cd mobility. In addition, the enrichment of Fe- and S-cycling-related bacterial taxa under the 100 nm treatment suggests potential microbial contributions to Cd immobilization and rhizosphere element cycling.

These findings indicate that 100 nm Se NPs at an appropriate application rate may provide a promising strategy for simultaneously reducing Cd accumulation and achieving Se biofortification in Cd-contaminated paddy soils. However, as discussed in Section 4.3, this study was conducted under controlled pot conditions using one soil type and one rice cultivar, and the microbial functions were mainly inferred from PICRUSt2-based functional prediction. Future studies should further verify the field-scale applicability, long-term environmental safety, microbial mechanisms, and Cd binding environments of Se NPs through multi-site field trials, omics-based approaches, isotope tracing, and in situ spectroscopic techniques.

## Figures and Tables

**Table 1:** Physicochemical properties of soil samples.

Physicochemical Property	Measured Value
pH	7.62
Organic matter (g/kg)	28.7
Total nitrogen (g/kg)	1.49
Total phosphorus (g/kg)	0.83
Total potassium (g/kg)	21.7
Alkali-hydrolyzable nitrogen (mg/kg)	142
Available phosphorus (mg/kg)	21.8
Available potassium (mg/kg)	215
Dehydrogenase activity (μg d^−1^g^−1^)	28.8
Urease activity (μg d^−1^g^−1^)	386
Catalase activity (μmol d^−1^g^−1^)	67
Cd (mg/kg)	0.790
Se (mg/kg)	0.321

## Data Availability

All data are contained within the article and Supplementary Materials.

## Supplementary Materials

The following supporting information can be downloaded at: https://www.mdpi.com/article/10.3390/nano16140897/s1. Figure S1. Synthesis and characterization of 50 nm, 100 nm, and 200 nm Se NPs. Figure S2. Dynamic light scattering (DLS)-determined particle size distributions and zeta potentials of nano-selenium particles with nominal sizes of 50, 100, and 200 nm. Figure S3. The content of SOM (A), AP (B), and AN (C) in rhizosphere soil at the maturation stage. Figure S4. The activity of DHA (A), UE (B), and CAT (C) in rhizosphere soil at the maturation stage. Figure S5. NMDS Ordination of Fungal (A) and Bacterial (B) communities in soils from different treatments. Figure S6. Rarefaction curves and species accumulation boxplots of fungi community (A,B) and bacteria community (C,D) in 100 nm Se NP treatment. Figure S7. Chao 1, Shannon and Simpson index of fungi community (A–C) and bacteria community (D–F) in 100 nm Se NP treatment. Figure S8. Relative abundance of soil microorganisms in 100 nm Se NP treatment. Figure S9. Graphical overview of the experimental design. Figure S10. SEM–EDS elemental mapping of 100 nm Se NPs. Figure S11. SEM–EDS point analysis of 100 nm Se NPs. Figure S12. Raman spectra of 50 (A), 100 (B), and 200 (C) nm Se NPs before and after spectral processing. Table S1. Composition and the content of Kimura solution. Table S2. XPS fitting parameters of Fe 2p spectra in root iron plaque under different treatments. Table S3. DLS-derived granulometric parameters of Se NPs suspensions in the liquid phase.
