# Supplementary material for: Size-Dependent Mechanism of Selenium Nanoparticles in Regulating Cadmium Accumulation in the Soil–Rice (Oryza sativa L.) System
Source: Nanomaterials (Basel). 2026 Jul 22;16(14):897. doi: 10.3390/nano16140897 (PMC13414602; doi:10.3390/nano16140897)
Supplement: Supplementary file 1 [file nanomaterials-16-00897-s001.zip › nanomaterials-4421308-supplementary.pdf]

Supplementary Materials

Size-Dependent Mechanism of Selenium Nanoparticles in Regulating Cadmium Accumulation in the Soil–Rice (*Oryza sativa* L.) System

Haonan Zhang [1,2], Zhangli Lu [3], Jianhao Tong [4], Jing Wang [1,2], Chendao Ruan [1,2], Ziming Xin [1,2], Zhenkun Deng [1,2] and Jiyan Shi [1,2,*]

[1] Department of Environmental Engineering, College of Environmental and Resource Sciences, Zhejiang University, Hangzhou 310058, China
[2] State Key Laboratory of Soil Pollution Control and Safety, Zhejiang University, Hangzhou 310058, China
[3] State Key Laboratory of Fluid Power and Mechatronic Systems, School of Mechanical Engineering, Zhejiang University, Hangzhou 310058, China
[4] College of Energy Engineering, Zhejiang University, Hangzhou 310027, China
* Correspondence: shijiyan@zju.edu.cn

Experiment S1 SeNPs synthesis and characterization.

The method described by Lin and Wang[1] was used to chemically synthesize SeNPs with sodium selenite (Na_2SeO_3) and sodium thiosulfate ($Na_2S_2O_3 \cdot 5H_2O$) in the pot experiment. The detailed steps for the chemical synthesis of SeNPs are shown in Figure 2-1. The morphology and microstructure of SeNPs were observed and measured using a field-emission scanning electron microscope (SEM, GeminiSEM 300, Germany) and a transmission electron microscope (TEM, H-7650, Hitachi, Japan). Subsequently, energy dispersive X-ray analysis (EDX) on the SEM cross-section was performed to analyze the elemental composition of selected areas. The particle size of SeNPs was determined by the method described by Hu et al..[2]

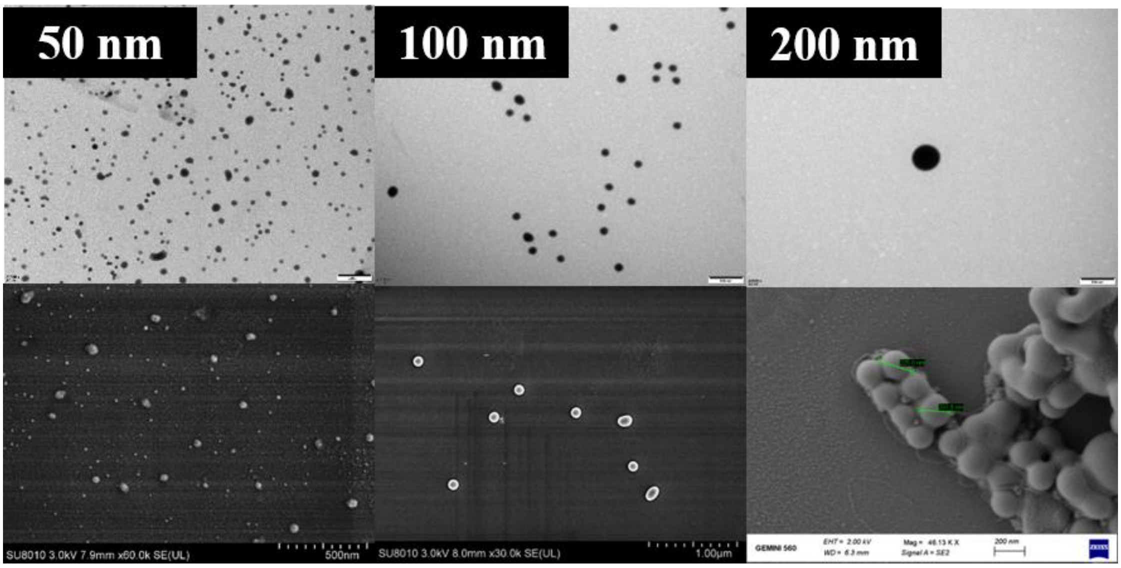

Figure S1. Synthesis and characterization of 50 nm, 100 nm, and 200 nm SeNPs. Scale bars: TEM images, 500 nm for all panels; SEM images, 500 nm, 1.00 μm, and 200 nm from left to right.

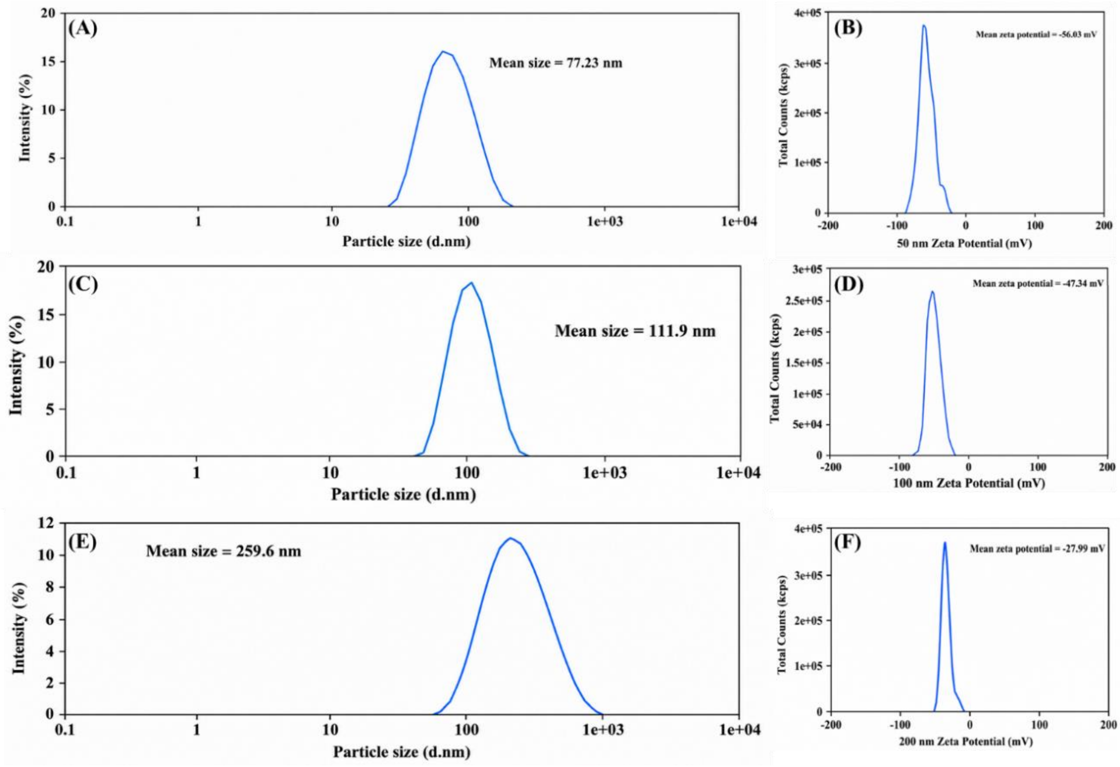

Figure S2. Dynamic light scattering (DLS)-determined particle size distributions and zeta potentials of nano-selenium particles with nominal sizes of 50, 100, and 200 nm. (A, C, and E) Particle size distributions of 50, 100, and 200 nm nano-selenium, respectively. (B, D, and F) Zeta potentials of 50, 100, and 200 nm nano-selenium, respectively.

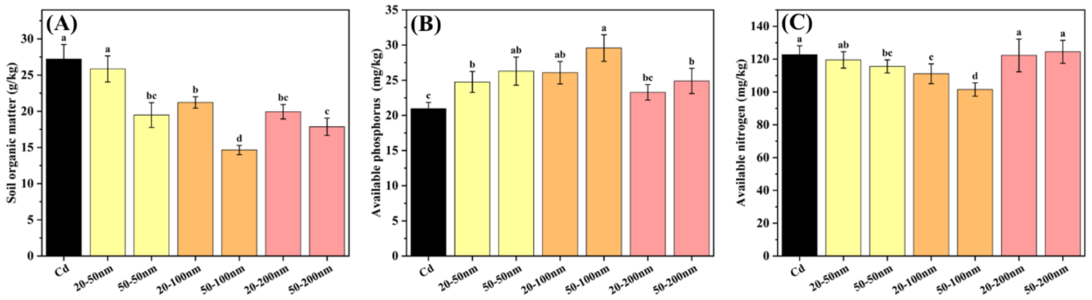

Figure S3. The content of SOM (A), AP (B), and AN (C) in rhizosphere soil at the maturation stage. Letters indicate significant differences between treatments ($p < 0.05$).

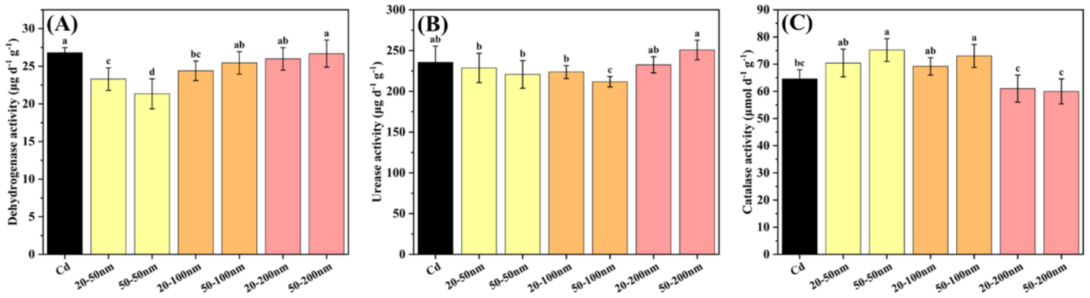

Figure S4. The activity of DHA (A), UE (B), and CAT (C) in rhizosphere soil at the maturation stage. Letters indicate significant differences between treatments ($p < 0.05$). .

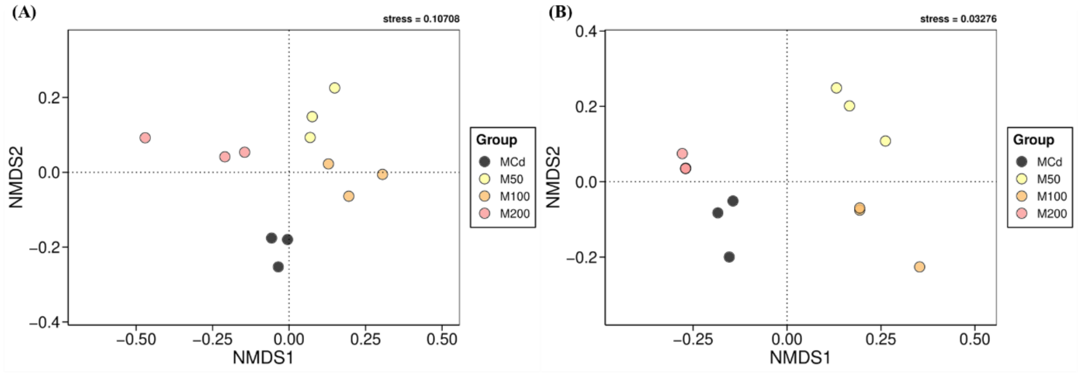

Figure S5. NMDS Ordination of Fungal (A) and Bacterial (B) Communities in Soils from Different Treatments, Stress < 0.2 indicates acceptable reliability of the NMDS analysis.

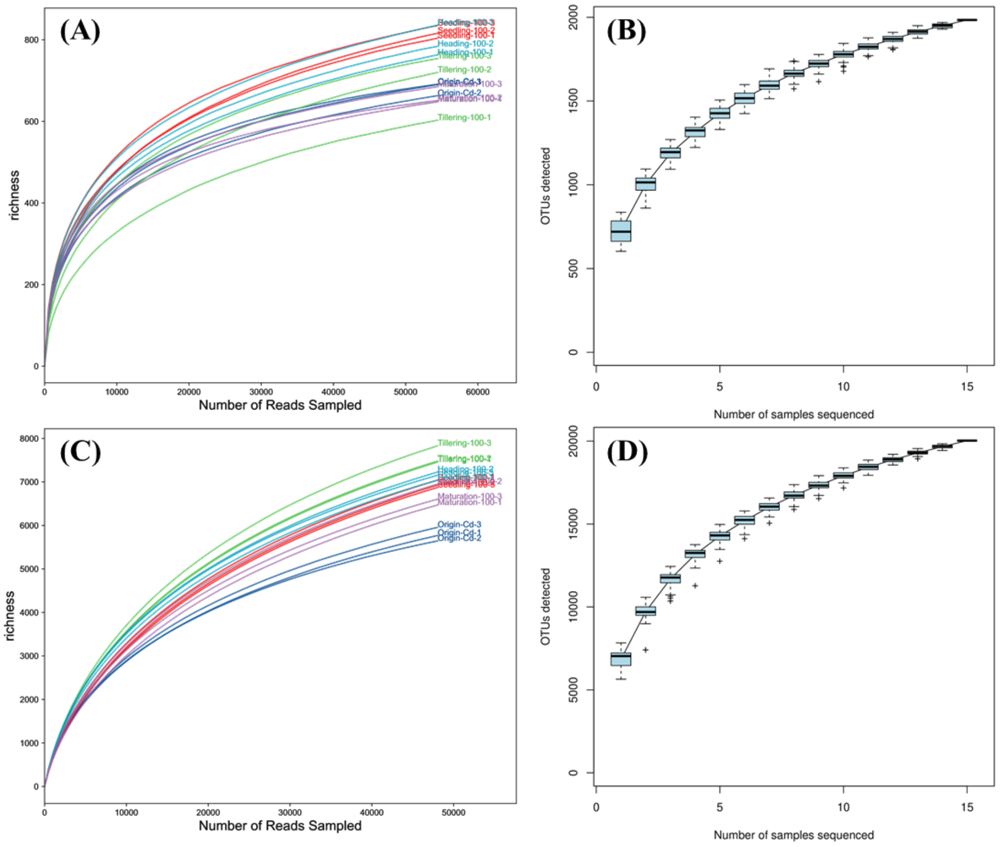

Figure S6. Rarefaction curves and species accumulation boxplots of fungi community (A, B) and bacteria community (C, D) in 100 nm SeNP treatment.

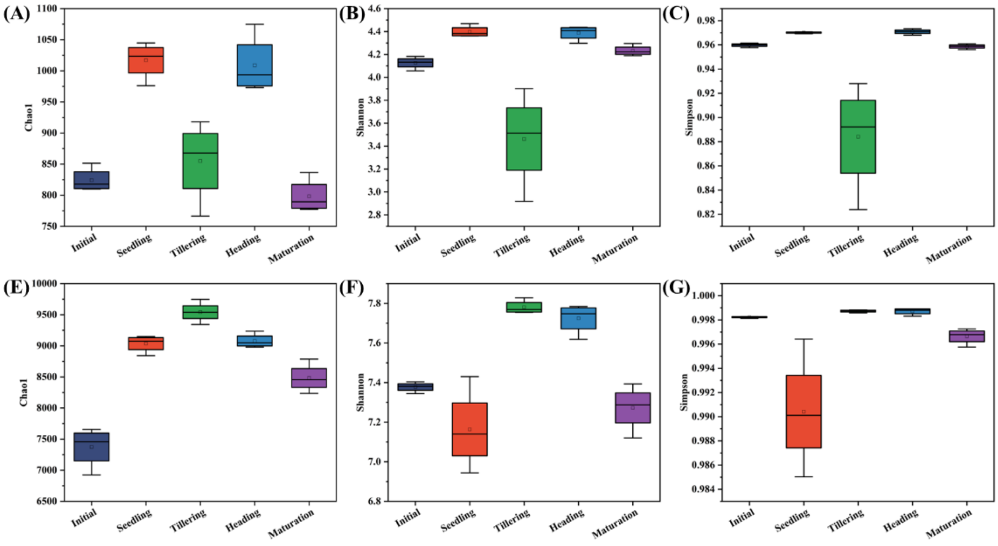

Figure S7. Chao 1, Shannon and Simpson index of fungi community (A–C) and bac-teria community (E–G) in 100 nm SeNP treatment.

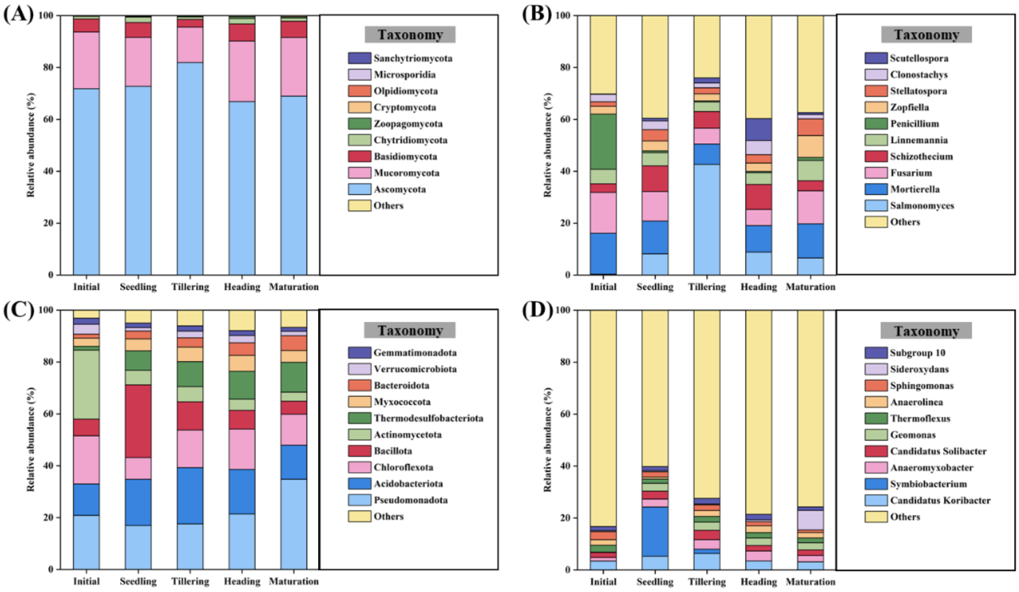

Figure S8. Relative abundance of soil microorganisms in 100 nm SeNP treatment. Relative abundance of fungal community at (A) phylum level (top 10) and (B) genus level (top 10); Relative abundance of bacterial community at (C) phylum level (top 10) and (D) genus level (top 10).

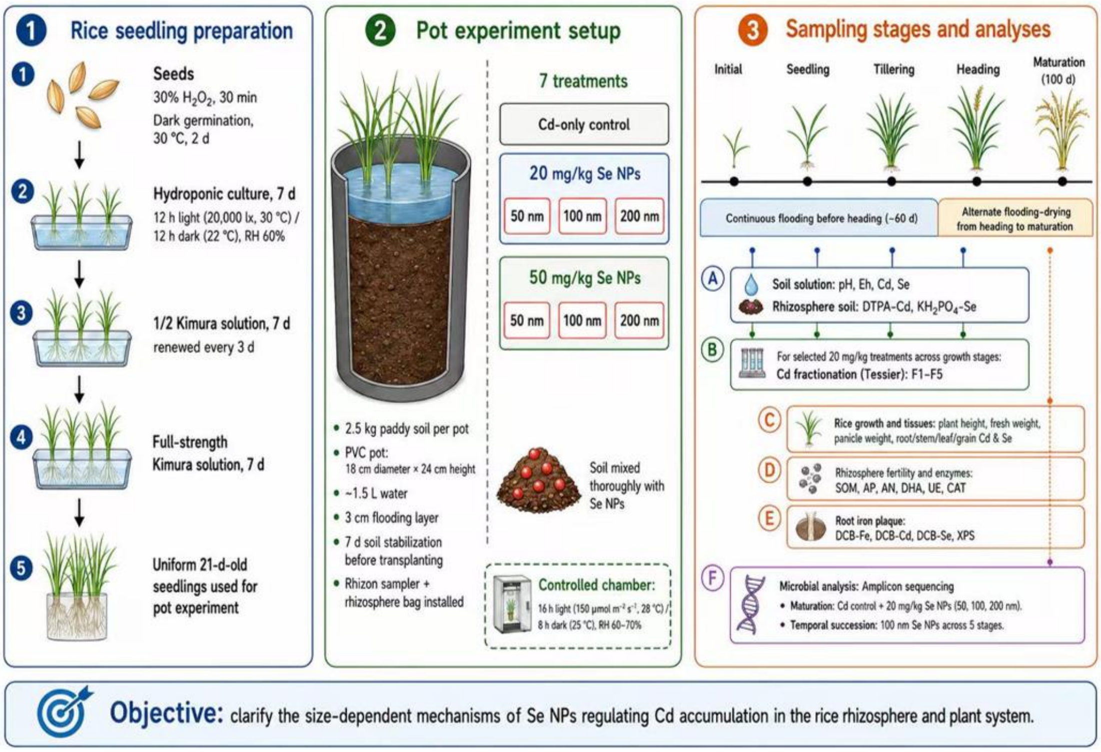

Figure S9. Graphical overview of the experimental design.

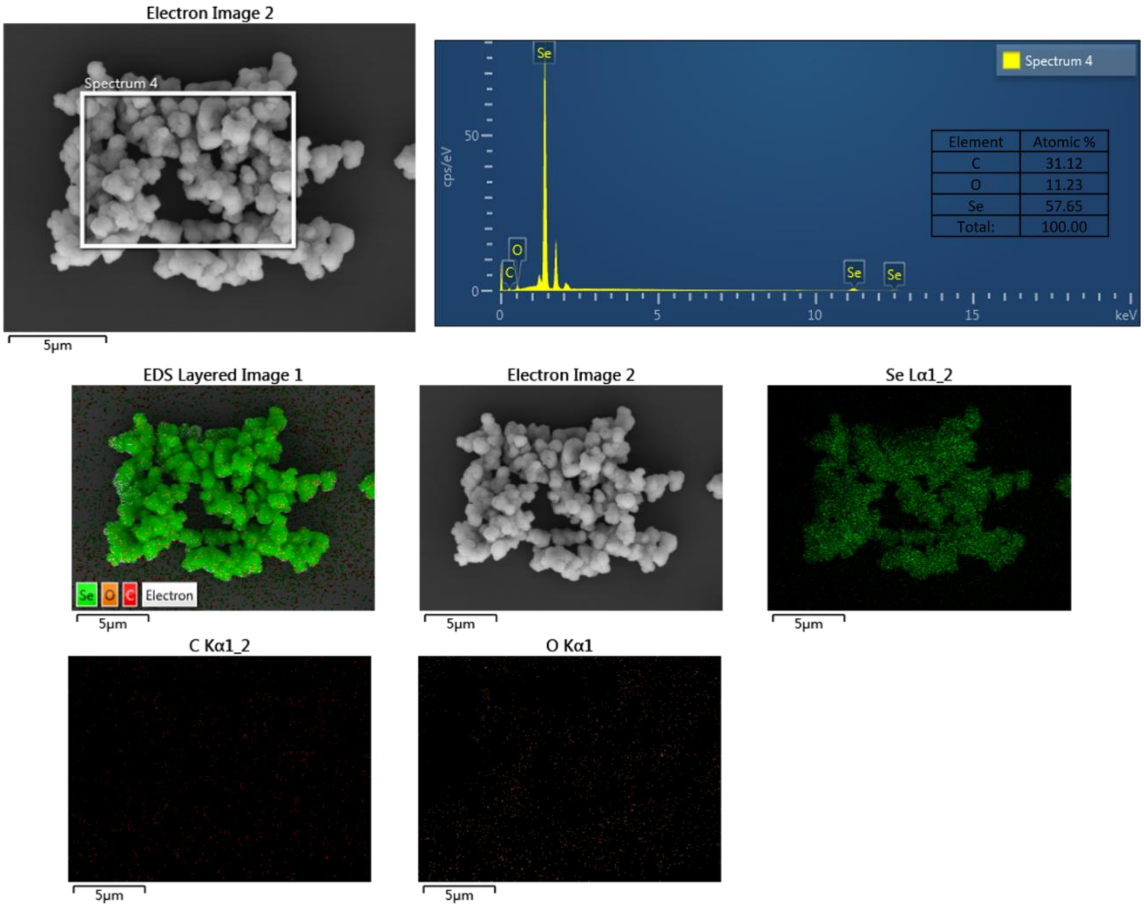

Figure S10. SEM–EDS elemental mapping of 100 nm Se NPs.

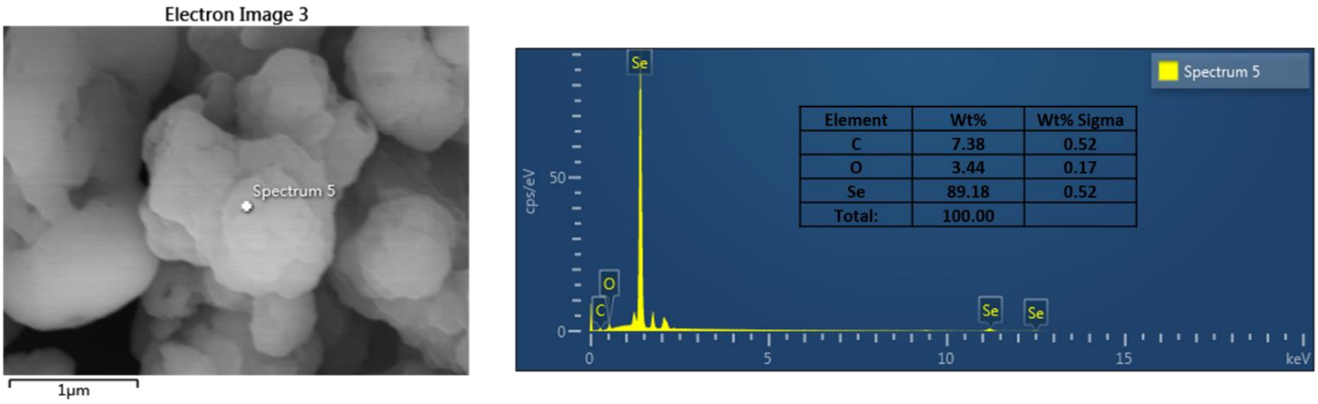

Figure S11. SEM–EDS point analysis of 100 nm Se NPs.

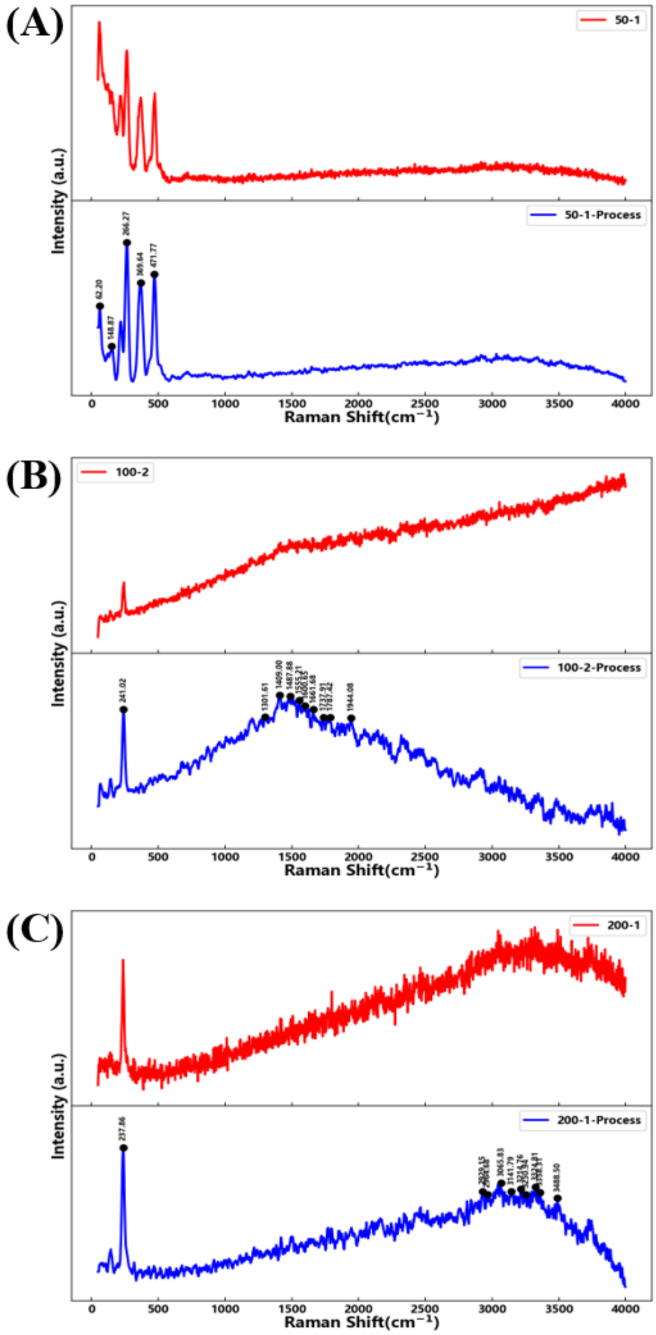

Figure S12. Raman spectra of 50 (A), 100 (B), and 200 (C) nm Se NPs before and after spectral processing. Characteristic Raman peak positions are labeled in the processed spectra.

Table S1. Composition and the content of Kimura solution.

Macro-element (mM)		Micro-element (μM)	
$(NH_4)_2SO_4$	0.18	$NaEDTA\text{-}Fe{\cdot}3H_2O$	20
$MgSO_4{\cdot}7H_2O$	0.27	$MnCl_2{\cdot}4H_2O$	6.7
KNO_3	0.09	H_3BO_3	9.4
$Ca(NO_3)_2{\cdot}4H_2O$	0.18	$(NH_4)_6Mo_7O_{24}{\cdot}4H_2O$	0.015

KH_2PO_4	0.09	$ZnSO_4{\cdot}7H_2O$	0.15
-	-	$CuSO_4{\cdot}5H_2O$	0.16

Table S2. XPS fitting parameters of Fe 2p spectra in root iron plaque under different treatments.

Sample	Element region	Peak assignment	Status	Binding energy (eV)	FWHM (eV)	Peak area (CPS·eV)	Proportion (%)	Work function (eV)
Cd	Fe 2p3/2	Fe 2p3/2 Fe(II)	Fe(II)	710.83	3.18	17134.06	61.67	4.2
Cd	Fe 2p3/2	Fe 2p3/2 Fe(III)	Fe(III)	712.88	3.37	10630.63	38.33	4.2
Cd	Fe 2p3/2	Fe 2p3/2 satellite	Satellite	716.11	3.37	5236.45		4.2
Cd	Fe 2p3/2	Fe 2p3/2 satellite	Satellite	720.25	3.37	3764.28		4.2
Cd	Fe 2p1/2	Fe 2p1/2 Fe(II)	Fe(II)	724.24	3.18	7516.36		4.2
Cd	Fe 2p1/2	Fe 2p1/2 Fe(III)	Fe(III)	725.96	3.37	4663.44		4.2
Cd	Fe 2p1/2	Fe 2p1/2 satellite	Satellite	728.69	3.37	3363.35		4.2
Cd	Fe 2p1/2	Fe 2p1/2 satellite	Satellite	733.49	3.37	2378.78		4.2
50 nm	Fe 2p3/2	Fe 2p3/2 Fe(II)	Fe(II)	710.6	2.87	16878.58	54.98	4.2
50 nm	Fe 2p3/2	Fe 2p3/2 Fe(III)	Fe(III)	712.6	3.37	13795.17	45.02	4.2
50 nm	Fe 2p3/2	Fe 2p3/2 satellite	Satellite	715.66	3.37	6274.43		4.2
50 nm	Fe 2p3/2	Fe 2p3/2 satellite	Satellite	719.33	3.37	5009.61		4.2
50 nm	Fe 2p1/2	Fe 2p1/2 Fe(II)	Fe(II)	723.71	2.87	7404.28		4.2
50 nm	Fe 2p1/2	Fe 2p1/2 Fe(III)	Fe(III)	725.4	3.37	6051.66		4.2
50 nm	Fe 2p1/2	Fe 2p1/2 satellite	Satellite	728.07	3.37	5932.42		4.2
50 nm	Fe 2p1/2	Fe 2p1/2 satellite	Satellite	733.1	3.37	3685.9		4.2
100 nm	Fe 2p3/2	Fe 2p3/2 Fe(II)	Fe(II)	710.57	2.83	18117.59	53.33	4.2
100 nm	Fe 2p3/2	Fe 2p3/2 Fe(III)	Fe(III)	712.62	3.37	15824.69	46.67	4.2
100 nm	Fe 2p3/2	Fe 2p3/2 satellite	Satellite	715.8	3.37	6452.49		4.2
100 nm	Fe 2p3/2	Fe 2p3/2 satellite	Satellite	719.63	3.37	5622.76		4.2
100 nm	Fe 2p1/2	Fe 2p1/2 Fe(II)	Fe(II)	723.82	2.83	7947.81		4.2
100 nm	Fe 2p1/2	Fe 2p1/2 Fe(III)	Fe(III)	725.55	3.37	6941.97		4.2
100 nm	Fe 2p1/2	Fe 2p1/2 satellite	Satellite	728.12	3.37	5130.94		4.2
100 nm	Fe 2p1/2	Fe 2p1/2 satellite	Satellite	732.84	3.37	3352.86		4.2
200 nm	Fe 2p3/2	Fe 2p3/2 Fe(II)	Fe(II)	710.5	2.99	14970.99	61.14	4.2
200 nm	Fe 2p3/2	Fe 2p3/2 Fe(III)	Fe(III)	712.7	3.37	9495.07	38.86	4.2
200 nm	Fe 2p3/2	Fe 2p3/2 satellite	Satellite	715.77	3.37	4264.13		4.2

200 nm	Fe 2p3/2	Fe 2p3/2 satellite	Satellite	719.63	3.37	3315.35	4.2
200 nm	Fe 2p1/2	Fe 2p1/2 Fe(II)	Fe(II)	723.88	2.99	6567.46	4.2
200 nm	Fe 2p1/2	Fe 2p1/2 Fe(III)	Fe(III)	725.92	3.37	4165.29	4.2
200 nm	Fe 2p1/2	Fe 2p1/2 satellite	Satellite	728.34	3.37	3174.38	4.2
200 nm	Fe 2p1/2	Fe 2p1/2 satellite	Satellite	732.9	3.37	2487.46	4.2

Table S3. DLS-derived granulometric parameters of Se NPs suspensions in the liquid phase.

Parameter	50 nm Se NPs	100 nm Se NPs	200 nm Se NPs
Z-average (nm)	68.84	102.7	145.8
PDI	0.1686	0.07139	0.419
Intercept	0.9699	0.9683	0.7931
Intensity peak mean (nm)	77.23	111.9	259.6
D10 (nm)	45.49	71.3	46.65
D50 (nm)	72.69	106.7	216.3
D90 (nm)	117.8	162.6	406.3
D95 (nm)	132.6	180.7	471.1

Reference

1. Lin, Z. H.; Wang, C. R. C., Evidence on the size-dependent absorption spectral evolution of selenium nanoparticles. *MATERIALS CHEMISTRY AND PHYSICS* **2005,** *92*, (2-3), 591-594.
2. Hu, T.; Li, H. F.; Li, J. X.; Zhao, G. S.; Wu, W. L.; Liu, L. P.; Wang, Q.; Guo, Y. B., Absorption and Bio-Transformation of Selenium Nanoparticles by Wheat Seedlings (Triticum aestivum L.). *FRONTIERS IN PLANT SCIENCE* **2018,** *9*.
